# Twist1 downregulation of PGC-1α decreases fatty acid oxidation in tubular epithelial cells, leading to kidney fibrosis

**DOI:** 10.7150/thno.71722

**Published:** 2022-05-01

**Authors:** Limin Liu, Xiaoxuan Ning, Lei Wei, Ying Zhou, Lijuan Zhao, Feng Ma, Ming Bai, Xiaoxia Yang, Di Wang, Shiren Sun

**Affiliations:** 1 Department of Nephrology, Xijing Hospital, Fourth Military Medical University, No. 127 Chang le West Road, Xi'an, Shaanxi, 710032, China.; 2 School of Medicine, Northwest University, 229 Taibai North Road, Xi'an, Shaanxi, 710069, China.; 3 Department of Geriatrics, Xijing Hospital, Fourth Military Medical University, No. 127 Chang le West Road, Xi'an, Shaanxi, 710032, China.

**Keywords:** Renal tubulointerstitial fibrosis, hypoxia, fatty acid metabolism, Twist1, pharmacological inhibition strategy

## Abstract

**Rationale:** A deficiency of fatty acid oxidation (FAO) is the metabolic hallmark in proximal tubular cells (PTCs) in renal fibrosis owing to utilization of fatty acids by PTCs as the main energy source. Lipid accumulation may promote lipotoxicity-induced pathological injury in renal tissue. However, the molecular mechanism underlying lipotoxicity and renal tubulointerstitial fibrosis (TIF) remains unclear. Twist1 has been identified to play an essential role in fatty acid metabolism. We hypothesized that Twist1 may regulate FAO in PTCs and consequently facilitate lipotoxicity-induced TIF.

**Methods:** We used hypoxia-induced Twist1 overexpression to incite defective mitochondrial FAO in PTCs, and used renal ischemia-reperfusion or unilateral ureteral obstruction to induce renal injury in mice. We used knockout cells, mice of Twist1, and Harmine to determine the role of Twist1 in FAO and TIF.

**Results**: Overexpression of Twist1 downregulates the transcription of PGC-1α and further inhibits the expression of FAO-associated genes, such as PPARα, CPT1 and ACOX1. Consequently, reduced FAO and increased intracellular lipid droplet accumulation in a human PTC line (HK-2), leads to mitochondrial dysfunction, and production of increased profibrogenic factors. Twist1 knockout mice with renal injury had increased expression of PGC-1α, which restored FAO and obstructed progression of TIF. Strikingly, pharmacological inhibition of Twist1 by using Harmine reduced lipid accumulation and restored FAO *in vitro* and *in vivo*.

**Conclusion:** Our findings suggest that Twist1-mediated inhibition of FAO in PTCs results in TIF and suggest that Twist1-targeted inhibition could provide a potential strategy for the treatment of renal fibrosis.

## Introduction

The kidney is an important metaboliser of biomaterials. Tubular epithelial cells (TECs) are one of most important cells in the kidney that maintain normal renal function. One characteristic of TECs is that they have a huge energy demand. The major source of adenosine triphosphate (ATP) in TECs, especially proximal tubular cells (PTCs), originates from the biochemical reaction of fatty acid β-oxidation (FAO) and not from glucose 1-4. Thus, reprogramming of lipid metabolism in PTCs under hypoxic stimulation is thought to be closely related to dysfunction of PTCs, and eventually result in the development of renal fibrosis 4, 5. Recently, it was reported that decreased capability of FAO in TECs caused intracellular lipid droplet accumulation, which results in lipotoxicity, represented as mitochondrial dysfunction combined with increased reactive oxygen species (ROS) production and decreased cellular ATP levels 6-9. More importantly, this may explain the phenomena of an increased apoptosis rate in PTCs and increased extracellular matrix production in the progression of renal fibrosis. However, more detailed molecular mechanisms linking lipotoxicity to renal fibrosis are still elusive.

Twist1, a member of the basic helix-loop-helix (b-HLH) transcription factor family 10, plays a critical role in cell cycle regulation and epithelial-mesenchymal transition (EMT), all of which are functionally associated with the development and progression of fibrotic diseases 11-13. In our previous study, we confirmed that Twist1 can promote the process of EMT in PTCs by reducing the expression levels of the epithelial markers E-cadherin and ZO-1, and enhancing the expression of the mesenchymal markers vimentin and α-smooth muscle actin (α-SMA) 14. In the unilateral ureteral obstruction (UUO)-induced renal fibrosis model, Twist1 deletion in PTCs inhibited the EMT process, maintained the integrity of cells, and restored cell proliferation and repair and regeneration of the kidney parenchyma, ultimately alleviating renal interstitial fibrosis 12. Additionally, Twist1 prolongs TGF-β-induced G2/M cell cycle arrest of TECs and leads to abnormal amplification of profibrogenic factors. Furthermore, Twist1 can attenuate NF-κB-induced inflammation responses and act as a critical regulator of metabolic adaptation in T helper cells involved in organ fibrogenesis 15,16. Strikingly, in a study of homeostasis of adipose tissues, it was demonstrated that Twist is involved in the regulation of fatty acid metabolism and consequently regulates the shift between white and brown adipose tissues 17-19. Therefore, we hypothesized that Twist1 may regulate FAO activation and further contribute to lipotoxicity-induced tubulointerstitial fibrosis.

In this study, we showed that hypoxia induces lipid accumulation and defective mitochondrial FAO with a subsequent profibrotic phenotype in PTCs. In this process, the Twist1-PGC-1α axis plays an essential role in the downregulation of functional genes involved in fatty acid metabolism. Importantly, in a renal fibrosis mouse model, we found that Twist1 deletion in proximal tubules led to upregulation of PGC-1α expression and prevented defective mitochondrial FAO activation. Twist1 is therefore functionally associated with renal fibrosis. To prove the potential clinical significance of our findings* in vitro* and *in vivo*, we utilized a pharmacological inhibition strategy to test the effect of Twist deletion in the treatment of renal fibrosis. We used the Twist inhibitor Harmine in UUO and unilateral ischemia reperfusion injury (UIRI)-induced renal fibrosis mouse models. We observed restoration of PGC-1α expression and a series of functional key genes regulating fatty acid metabolism, such as peroxisome proliferator-activated receptor alpha (PPARα), CPT1, and peroxisomal acyl-coenzyme A oxidase 1 (ACOX1), and further confirmed inhibition of lipid accumulation in PTCs. Taken together, these data suggest that the hypoxia-induced Twist1-PGC-1α axis regulates mitochondrial lipid metabolism and contributes to progressive renal interstitial fibrosis. More importantly, it sheds light on the avenue to explore a potential therapeutic strategy for the treatment of renal fibrosis.

## Materials and methods

### Cell culture and treatment

The human proximal PTC line (HK-2) was purchased from BeNa Culture Collection (Peking, China, BNCC33801) 14 was cultured in DMEM (Gibco, Invitrogen, Grand Island, NY, USA) containing 10% fetal bovine serum (Gibco, Invitrogen, Grand Island, NY, USA) supplemented with 5 mg/mL insulin, 5 mg/mL transferrin, 5 ng/mL selenium, 36 ng/mL hydrocortisone and 4 pg/mL triiodothyronine. The cells were cultured for 0, 6, 12, 24, 48 or 72 h under hypoxic conditions (1% O_2_, 5% CO_2_, 37 °C) in an incubator (Precision Scientific, Winchester, VA, USA) and under normoxic conditions (21% O_2_, 5% CO_2_) for 0 or 48 h. As required, cells were treated with 20 μmol/L, 40 μmol/L Harmine or physiological saline water for 48 h 20.

### Animal model

All mouse work was performed in accordance with the animal use protocol approved by the Animal Care and User Committee of the Fourth Military Medical University (Approval number: KY20183109-1). Male C57BL/6J mice (8/group) aged 6-8 weeks and weighing 20-25 g were obtained from the Fourth Military Medical University Laboratory Animal Center (Xi'an, China). Homozygous Twist1 (flox/flox) mice (B6; 129S7-*Twist1^tm2Bhr^*/Mmnc) were purchased from The Mutant Mouse and Research Center-University of North Carolina (RRID: MMRRC_016842-UNC), and mice PEPCK-Cre mice were donated by Professor Dong Zheng (Medical College of Georgia at Augusta University). PEPCK promoter that was used to drive Cre expression in proximal tubular cells and established in Dr. Volker Haase's laboratory as described previously 21. Twist1^ (flox/flox)^ mice were crossed to PEPCK-Cre mice to produce Twist1^(flox/flox)^ X^(Cre)^Y as depicted in the breeding protocol in Figure S1A. The generated mice were heterozygous for the Twist1 floxed allele. These mice were crossbred to generate offspring with different littermates [Twist1^(flox/flox)^ X^(Cre)^ Y, Twist1^(+/+)^X^(Cre)^ Y, Twist1^(flox/flox)^ XY, Twist1^(+/+)^ XY]. Twist1^(flox/flox)^ X^(Cre)^ Y mice were Twist1-deleted in renal proximal tubules (named PT-*Twist1*^-/-^). These mice and wild-type littermates (male, 6 to 8 weeks old) were used for ischemic renal injury experiments. Genotyping was performed by PCR assay using DNA extracted from the mouse tail and using the following primers: Cre transgene, sense: 5'- GCGAACATCTTCAGGTTCTG -3'; antisense: 5'- CTTCAGGTTCTGCGGGAAACC -3'; Twist1 floxed, sense: 5'- GCTGACCATGGCTATGATCC -3'; antisense: 5'- CCGTGCTGACCATGGCTATG -3'.

UUO model: Mice were anesthetized by intraperitoneal injection of 5% pentobarbital sodium (50 mg/kg). Incision (1.5 cm) in the upper left quadrant was made, and the left ureter was sutured and ligated with silk thread. In sham-operated mice, the left ureter was not ligated; other steps were the same 22. Harmine (Med Chem Express, 10 mg/kg or 20 mg/kg body weight) was injected intraperitoneally into mice from Day 0 to Day 14 after UUO operation. The sham groups were injected with the same volume of PBS (Harmine solvent).

UIRI model: After left side incision, the left renal pedicle was clamped with a microvascular clamp at 37 °C for 30 min, and then the clamp was released and re-perfused after ischemia. The sham-operated mice underwent the same process but did not clamp the renal pedicle 23. On the 14^th^ day of UUO and the 28th day of UIRI, the kidneys were removed for histological, RNA and protein analysis.

### Kidney biopsies

The study was approved by the Xijing Hospital's Protection of Human Subjects Committee (Approval number: KY20163162-1), and informed consent was obtained from all patients and volunteers. A total of 20 renal biopsy samples were collected from patients with CKD treated in Xijing Hospital from January 2018 to December 2020. The selected tissue samples had at least 10 glomeruli for further study. Histological examination was carried out in the Department of Pathology of Xijing Hospital, and the corresponding clinical information was collected from the patient's medical records. Patients with severe infections, immunodeficiency, liver disease and malignant tumors were excluded from this study. Nine samples of control kidney tissue were taken from the Department of Urology of Xijing Hospital from patients who underwent nephrectomy or partial nephrectomy because of renal injury or partial resection.

### Measurement of mitochondrial FAO

Seahorse XF Analyzer (Agilent Technologies Co. Ltd., USA) was used to measure mitochondrial FAO according to the procedures provided by the manufacturer. Cells treated with normoxia, hypoxia or transfected with Twist1 plasmids were inoculated in XF24 cell culture microplates at a dose of 1.0×10^4^ cells per well. FAO activity was evaluated by measuring the difference in maximum respiratory volume among different treatment groups. The function of mitochondrial FAO was evaluated by the oxygen consumption rate (OCR) at baseline and with the addition of oligomycin (1 µM, a blocker of ATP synthesis on the mitochondrial intima that forces cells to rely on glycolysis to provide ATP, Seahorse Bioscience), FCCP (1 μmol/L, carbonyl cyanide-4-[trifluoromethoxy] phenylhydrazone that uncouples electron transport during ATP synthesis and restores mitochondrial membrane potential differences) and rotenone (1 μmol/L, an inhibitor of ATP synthase that inhibits FAO, Sigma) to HK-2 cells at 24-min intervals 4,6.

### Oil Red O staining

ORO staining was performed on normoxic and hypoxia-treated HK-2 cells and pcDNA-Twist1, pcDNA-PGC-1α, siTwist1 or Harmine-transfected HK-2 cells. The ORO-polyethylene glycol method described by Kinkel *et al.* was used to stain the cells 24. The staining procedure followed the method established by Mehlem *et al* 25. ORO staining was performed on 12 mm thick unfixed, freshly frozen animal kidneys and kidney tissues of patients with CKD. Cells and samples stained with Oil Red O (0.5% in isopropanol) for 20 min. Computer-aided density image analysis was used for quantitative evaluation of ORO staining. Lipid droplets (LDs) were counted on an EVOSⓇFL Auto Cell Imaging System (Thermo Scientific) and reported as rate of clustered and dispersed LDs. LDs per 100 cells were analyzed. The distribution of lipid droplets was scored as densely clustered (Clustered), and fully dispersed (Dispersed) as described by Marcinkiewicz A *et al.* 26. Data are the average of 100-cell counts for each of three independent experiments.

### Quantitative RT-PCR

TRIzol (Invitrogen, Inc.) was used to crack cells and conduct RT-PCR as previously described 14, 27. All PCR experiments were performed in triplicate. The primer sequences and full gene names are shown in Table S1. The 2^-ΔΔCT^ CT method was used to standardize the relative gene expression to β-actin 28.

### Western blotting analysis

Western blotting was carried out as previously described 14. Briefly, proteins in cells or kidney lysates were quantified using a BCA protein analysis kit (Thermo Fisher Science). A total of 60 μg of protein lysate was electrophoresed on an 8% SDS-polyacrylamide gel and imprinted on a nitrocellulose membrane (Millipore, Bedford, MA, USA). The membrane was incubated with the primary antibody overnight at 4 °C, and then the anti-rabbit or anti-mouse secondary antibody coupled with horseradish peroxidase conjugation was incubated at room temperature. An enhanced chemiluminescence system (Amersham Pharmacia Biotech) and enhanced chemiluminescence agent (Zeta-LIFE) were used for visual detection, and Western blot detection of β-actin was used as an internal sample control.

### Plasmid constructs and transfection and the dual-luciferase reporter gene assay

The recombinant sense expression vector and small interference RNA expression vector of Twist1 were constructed as previously described 14. The human PGC-1α gene was amplified from hypoxia-treated HK2 cells and subcloned into the eukaryotic expression vector pcDNA3.1. The specific targeting sequence of PGC-1α siRNA was inserted into the pSilencer3.1U6neo siRNA expression vector (Genecem, China). The scrambling sequence was used as a control (pSilencer3.1 transfection). According to the instructions of the manufacturer, Twist1 plasmid si-Twist1 and/or PGC-1α plasmid were transfected with Lipofectamine 3000 (Invitrogen). The sequences above are shown in Table S2-5.

Dual-luciferase reporter gene detection: the promoter vector containing PGC-1α of TBS1 was constructed, that is, TBS2 was mutated (CATGTG into CACCTG); the promoter vector of PGC-1α containing TBS2 was constructed, that is, TBS1 was mutated (CATGTG into CACCTG). PcDNA-Twist1 (20 ng), pcDNA-PGC-1α (20 ng) and/or reporter plasmid pGL-CMV were cotransfected with Lipofectamine 3000 (Invitrogen). Luciferase activity was determined 48 h after transfection. Luciferase activity was detected by the ModulusTM Single Tube Detection System (Turner BioSystems) and Steady-Glo Luciferase Reporter Assay Kit (Promega). Each independent experiment was repeated three times.

### Chromatin immunoprecipitation analysis

HK-2 cells were fixed with 1% paraformaldehyde, and F550 microtip cell sonar was used to splice and separate the chromatin of the nucleus (Fisher Scientific, Morris Plains, NJ). After centrifugation, the supernatant was collected and incubated with anti-Twist1 antibody or control IgG, and Protein A sepharose was added for overnight incubation, followed by elution of the immune complex. The complex was treated with RNase and proteinase K, and DNA was extracted with phenol/chloroform. DNA was precipitated, washed, dried and suspended in water for PCR analysis. The primers used in this analysis spanned 173 bp around the first possibility of the TBS located -1154 bp from the translation start site (sense, 5'- CTAGCTAGCTGATGGACACAAAGCAGCCT -3' and antisense, 5'- CCGCTCGAG AGTGCTGAGAATTCGCCCAA -3'), and the second possibility of the Twist1-binding site located -1104 bp from the translation start site within the PGC-1α promoter (sense, 5'-AGTCCTCC AAACCAAATCTGTCT -3' and antisense, 5'- CAGTCTTTCAAGCGCACTGT -3').

### Histology and histopathology

Kidneys were fixed in 10% neutral buffered formalin and embedded in paraffin. 5 µm sections were cut for hematoxylin-eosin (H&E) and Masson trichrome staining (Leica). Masson trichrome staining for collagen was performed using 2% aniline blue and 0.7% acid fuchsin and counterstained with Weigert's ematoxylin (Beijing Zhongshan Jinqiao Biotechnology Co., LTD, China). The extent of renal injury was estimated using morphometric assessment of tubular damage and interstitial fibrosis. To evaluate the degree of damage to renal tubules in mice, eight 400× visual fields were randomly selected from each section, and the number of healthy renal tubules was manually counted using the Adobe Photoshop counting tool. Tubules were defined as healthy when the dimension, structure, relative nucleus-cytoplasm configuration, brush border, and basal membrane integrity were similar to those of healthy tubules. For the analysis of interstitial fibrosis of mice in each group, eight 400× fields were randomly selected from each Masson trichrome staining renal tissue section, and the degree of interstitial fibrosis was manually evaluated by Adobe Photoshop for grid cross analysis. A Leica DM 1000 LED microscope and MC120 HD microscope camera were used, and Las V4.4 software (Leica) was used to collect representative images.

### Immunohistochemistry and immunocytochemistry

The immunohistochemical method was to fix 3- to 5-μm thick animal tissue and kidney biopsy sections of CKD patients on glass slides for dewaxing, rehydration, permeation and sealing. The slide and the primary antibody were incubated overnight at 4 °C. The slices were then incubated with appropriate biotinylated secondary antibodies and visualized. Non-immunized goat IgG or rabbit IgG was used as a control.

HK-2 cells were inoculated on aseptic slides, and immunofluorescence cell detection was performed by using a protocol established previously 14. The cells were incubated with primary antibody at 4 °C overnight. The slides were then incubated with secondary antibodies (FITC-labeled goat anti-mouse antibody or anti-rabbit IgG) at room temperature for 1 h. The nuclei were stained with 4', 6-diamidino-2-phenylindole (DAPI), and the sections were examined by laser confocal scanning microscopy.

### Antibodies

The following antibodies were used in this study: rabbit polyclonal antibody: Twist1 (Abcam, Cambridge, UK, Cat# ab175430, RRID:AB_883290, for Western blotting; Proteintech, Rosemont, IL, USA, Cat# 18125-1-AP, RRID:AB_10860102, for immunohistochemistry and immunofluorescence), HIF-1α (Beijing Biosynthesis Biotechnology Co., Ltd., China, Cat# bs-20399R), α-smooth muscle actin, PGC-1α (Abcam, Cat# ab54481, RRID:AB_881987), PPARα (Abcam, Cat# ab8934, RRID:AB_306869), COL1A1 (Abcam, Cat# ab88147; RRID:AB_2081873), COL4A1 (Abcam, Cambridge, UK, Cat# ab6311, RRID: AB_305414), β-actin (Santa Cruz Biotechnology Cat# sc-47778, RRID:AB_626632), and ACOX1 (Abcam, Cat# ab59964, RRID:AB_940106). Mouse polyclonal antibodies: Fibronectin (Abcam, Cat# ab2413; RRID: AB_2262874), CPT1 (Abcam, Cat# ab128568, RRID: AB_11141632).

### RNA-Seq experiments

RNAs from sham and UUO kidney cortex (the renal cortex were cut with laser capture microdissection [Thermo Scientific] to obtain renal tubules) were extracted separately from three randomly selected individual samples (as one biological replicate). Three biological replicates were used for each group. Total RNA was extracted using the RNeasy Plus Kit (Qiagen, Hilden, Germany) according to the manufacturer's instructions and RNA concentrations were determined using a NanoDrop 1000 (Thermo Fisher Scientific). RNA-Seq, data generation and normalization were performed on the Illumina Cluster Station and Illumina HiSeq 2000 System at Guangzhou Genedenovo Corp. The RNA-seq data described herein have been deposited in the National Center for Biotechnology Information Sequence Read Archive (SRA) under the accession code SRP322856, (https://www.ncbi.nlm.nih.gov/sra/PRJNA735313).

### Statistical analysis

Each experiment was repeated at least 3 times. All statistical analyses were performed using one-way ANOVA with SPSS 19.0. Comparisons between groups were made using a 2-tailed unpaired Student's *t*-test. *P* < 0.05 was considered significant.

## Results

### Triglyceride accumulation increases in PTCs exposed to hypoxia and is present in kidneys with tubulointerstitial fibrosis

**Figure 1:**
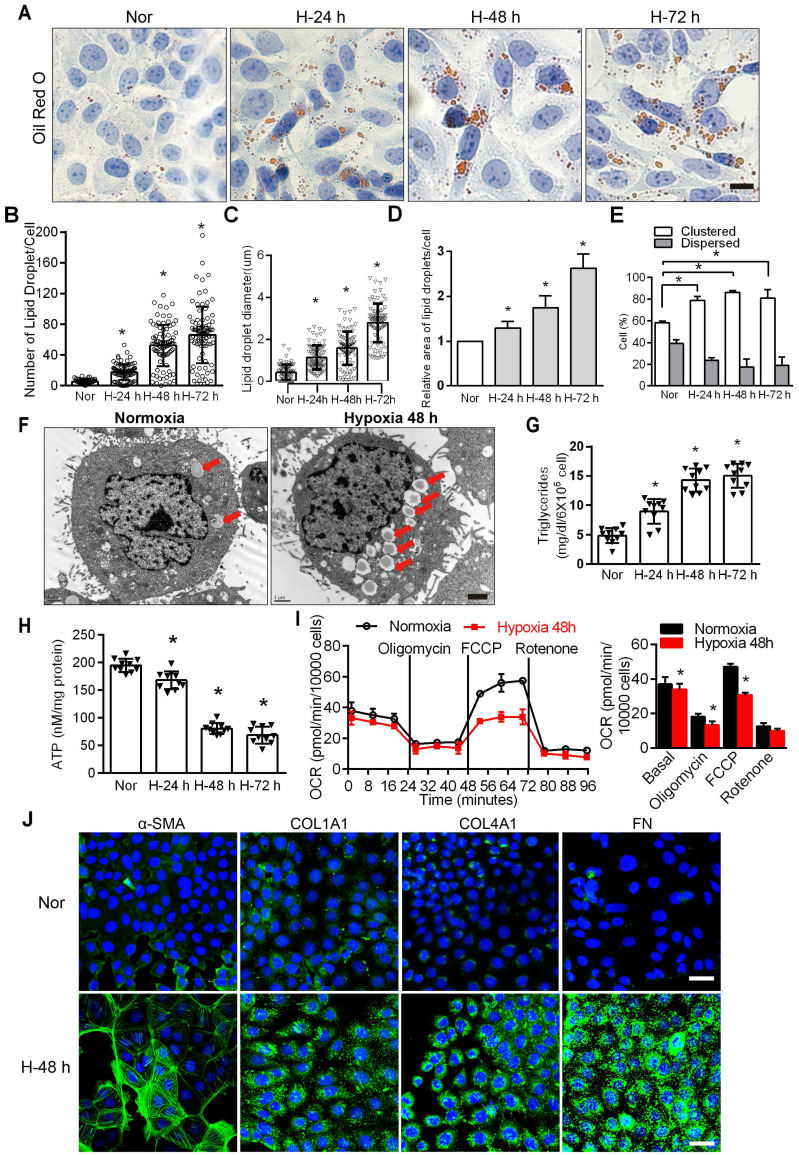
** Hypoxia-induced defective FAO and up-expression of fibrosis factors in PTCs. A.** Lipid droplets were detected by ORO staining, Scale bar, 25 µm, and **F** electron microscope. Red arrows indicate lipid vacuoles. Bars = 2 µm. **B.** High-Content Screening Studio Cell Analysis Software analyzed the number of lipid droplets in the HK-2 cells treated with hypoxia and normoxia. **C and D.** The relative diameter and relative area of lipid droplets in cells were analyzed by Image J software. E. Scores indicate the distribution of lipid droplets (Dispersed or Clustered) in cells with different hypoxia time were analyzed by EVOS®FL Auto Cell Imaging System and Image J software. *P < 0.05 compared with the normoxic group. The patterns depicted densely clustered (Clustered) and fully dispersed (Dispersed) 26. Data are the average of 100-cell counts for each of three independent experiments. **G.** Detected triglyceride content of HK-2 cells extracted from normoxic (Nor) and hypoxia for 24 h (H-24 h), 48 h (H-48 h) and 72 h (H-72 h), respectively. *P < 0.05 compared with normoxic group. **H.** The content of ATP in HK-2 cells under normoxic (Nor) and hypoxia for 24 h (H-24 h), 48h (H-48 h) and 72 h (H-72 h) was detected. *P < 0.05 compared with the normoxic group. **I.** OCR in HK-2 cells exposed to hypoxia for 48 h or normoxia cells. Representative traces are shown on the left, and the summary data analyzed for 24 wells from 3 independent experiments are shown on the right. Where indicated, oligomycin (1 µM), FCCP (1 µmol/L) and rotenone (1 µmol/L) were added. **J.** Immunofluorescence analyses α-SMA, COL1A1, COL4A1 and FN (fibronectin) expression in HK-2 after 48 h of hypoxia (green), with DIPA (delta-interacting protein A) shown in blue. Scale bar, 10 µm. *P < 0.05 throughout the figure by unpaired Student's *t* test. All data throughout the figure are shown as the mean ± SEM. Each independent experiment was repeated three times.

FAO is the main energy supply for TECs 1, 29, suggesting that disordered fatty acid metabolism of PTCs induced by chronic hypoxia may be key to the induction of tubular cell injury. The accumulation of intracellular lipid droplets was significantly increased under hypoxic conditions in a time-dependent manner (Figure 1A). In hypoxic cells, the number, relative diameter, relative area, and percentage of lipid droplet accumulation in cells all increased significantly and were significantly higher than those observed in normoxic cells (^*^P < 0.05) (Figure 1B-E). Electron microscopy showed obvious lipid droplets in the cells treated with hypoxia for 48 h (Figure 1F). The triglyceride content increased (Figure 1G), and ATP decreased significantly in HK-2 cells exposed to hypoxic conditions (^*^P < 0.05) (Figure 1H). We examined the OCR of the HK-2 cell line added substrate inhibitors, long-chain fatty acid oxidation was inhibited by etomoxir, glucose/pyruvate was antagonized by UK5099, glutamate synthesis was suppressed by Bis-2-(5-phenylacetamido-1,3,4-thiadiazol-2-yl) ethylsulfide (BPTES). The increase in OCR (especially maximum respiration) was sensitive to etomoxir, confirming its specificity. OCR was unchanged after UK5099 and BPTES treatment (Figure S2). Consistent with previous reports 29, 30, we found that FAO is the key contributor to intracellular ATP levels in PTCs. We added oligomycin, a blocker of ATP synthesis on the mitochondrial intima that forces cells to rely on glycolysis to provide ATP; FCCP (carbonyl cyanide-4-[trifluoromethoxy] phenylhydrazone), which uncouples electron transport during ATP synthesis and restores mitochondrial membrane potential differences; and rotenone, an inhibitor of ATP synthase that inhibits FAO, to HK-2 cells at 24 min intervals 4, 6. The results showed that the cells treated with hypoxia had lower baseline OCRs and FCCP-induced decreases in OCR, indicating that the fatty acid metabolism activity of PTCs cultured under hypoxic conditions was reduced (Figure 1I). Furthermore, compared with normoxia, hypoxia induced a significant increase in the expression of fibrosis factors (α-SMA, Collagen I [COL1A1], Collagen IV [COL4A1], Fibronectin [FN]) (^*^P < 0.05), as tested by immunofluorescence in HK-2 cells after hypoxia (48 h) (Figure 1J).

**Figure 2:**
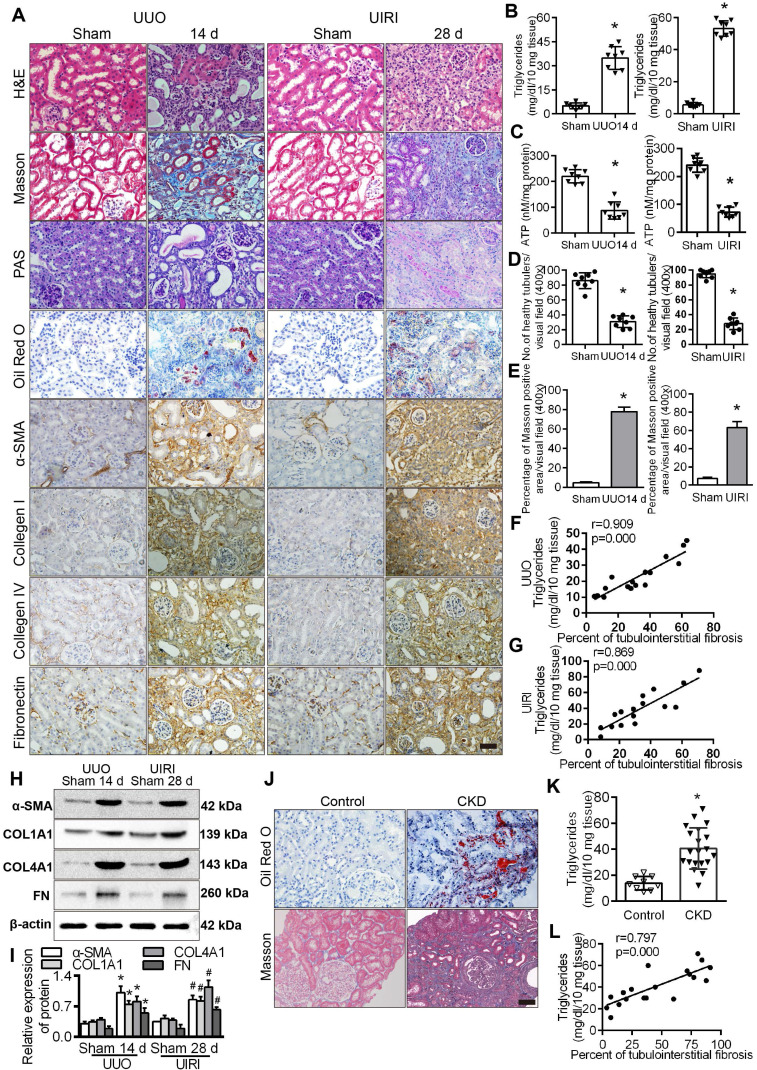
** Tubule epithelial FAO defects in CKD and mouse fibrosis models. A.** The expression of α-SMA, COL1A1, COL4A1 and FN was detected in the kidneys of the sham-operated group, UUO after 14 days and UIRI after 28 days by Immunohistochemical (400×). Scale bar, 50 µm. n = 8, in each group. **B.** The triglyceride content in the renal tissues of the sham-operated group and the UUO and UIRI groups was detected by a triglyceride test kit. n = 8/group. **C.** Analysis of ATP in the kidney tissues of the sham, UUO and UIRI groups. n = 8/group. **D.** Number of healthy tubules, on the basis of H&E. n = 8/group. **E.** Tubulointerstitial fibrosis were evaluated by Masson's trichrome staining. n = 8/group.** F and G.** Analysis of the correlation between the degree of renal interstitial fibrosis and triglyceride in renal tissue of UUO and UIRI groups. n = 8/group. **H and I.** Levels of fibrosis factor α-SMA, COL1A1, COL4A1 and FN in the kidney tissues of the sham operation group, UUO group and UIRI group were detected by Western Blots. *P < 0.05 compared with the control group, n = 8/group for panels. **J.** Kidney tissue from patients with CKD (n = 20) and controls (n = 9) were stained with ORO and Masson (400×). Scale bar, 50 µm. **K.** Detected triglyceride in kidney samples from controls and patients with CKD. **L.** Analysis of the correlation between the degree of renal interstitial fibrosis and triglyceride in renal tissue of patients with CKD. *P < 0.05 compared with the control group.

We observed that lipid accumulation, triglyceride content and the expression of fibrosis factors in the kidneys of UUO and UIRI model mice were significantly higher than those in the sham operation group (Figure 2A-B), while ATP content was significantly lower (Figure 2C). Analyses of fibrotic kidneys following H&E, and Masson staining revealed decreased tubular health and a higher degree of interstitial fibrosis in the UUO and UIRI mice kidneys (Figure 2D-E), and the level of triglycerides was directly proportional to the percentage of TIF in renal tissue (Figure 2F-G). The level of fibrosis factors in renal tissue of UUO and UIRI mice was significantly higher than that of sham mice (Figure 2H-I). There was significant fibrosis and lipid accumulation in renal interstitial tissue from patients with CKD (Figure 2J) and a significant increase in triglyceride content compared with normal renal tissue (Figure 2K), which was positively correlated with the degree of renal interstitial fibrosis (Figure 2L). Patient characteristics are summarized in Table S6. These results suggested that hypoxia causes a disturbance of FAO in PTCs, which is closely related to the progression of fibrosis.

### Genome-wide transcriptome analyses of mouse models (UUO) of fibrosis showed enrichment in metabolism

**Figure 3:**
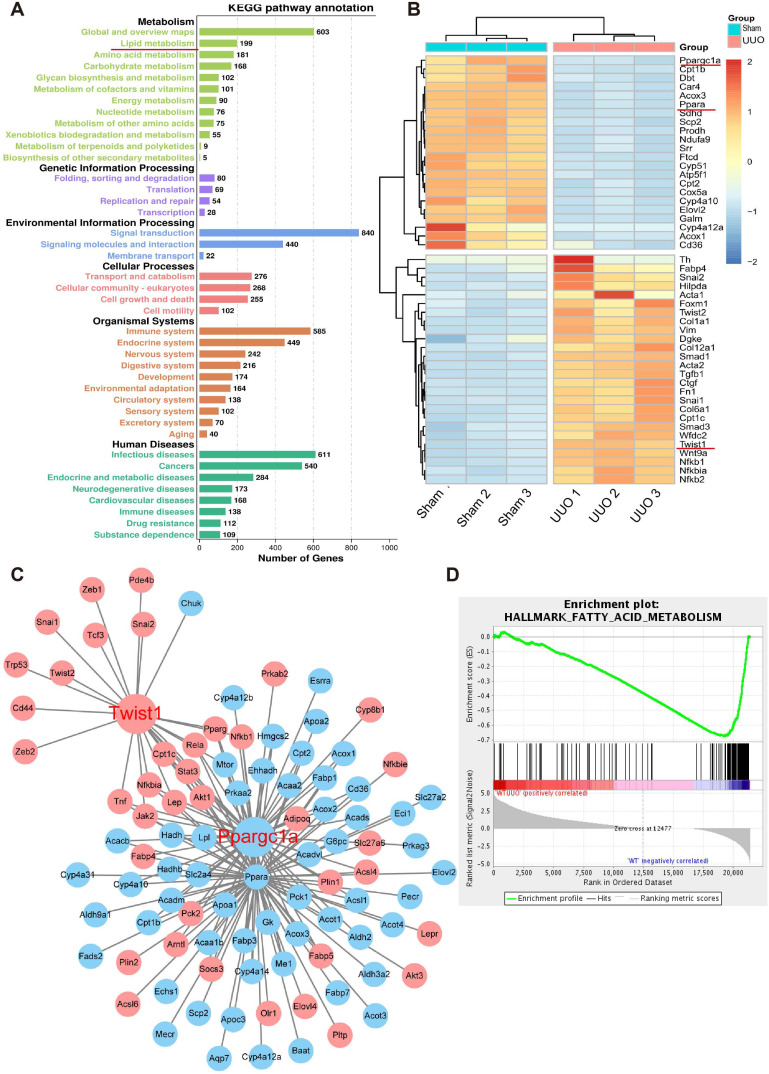
** Genome-wide transcriptome analyses of mouse models (UUO) of fibrosis. A.** UUO mice renal cortices were cut with laser capture microdissection (Thermo Scientific) to obtain renal tubules. GO term analysis of the number of genes that differed between the sham and UUO kidney tissue in different pathways. **B.** Heatmap analysis of the expression of transcripts related to fatty acid metabolism. **C.** Protein-protein interaction network of differentially expressed genes. Network nodes represent proteins, blue and red nodes represent down- and up-regulated genes, and lines indicate protein-protein associations. **D.** Gene set enrichment analysis highlighting strong enrichment for the FAO pathway in samples obtained from the kidneys of mice with UUO.

To further study the key molecules involved in renal interstitial fibrosis, we collected and analyzed changes at the genomic transcription level in UUO mice (n = 9) and a normal control group (n = 9). A total of 21,368 genes were analyzed by RNA-seq transcriptome sequencing, and 7210 genes with significant differences were identified. Gene Ontology (GO) term analysis visualized by the GO plot showed that the differentially identified genes were significantly enriched in metabolism (Figure S3). Cell metabolism in the UUO sample was abnormal, and the GO enrichment analysis emphasized significant changes in fatty acid, amino acid, and carbohydrate metabolism (Figure 3A). Further heat map analysis of gene expression showed that the levels of genes related to fatty acid metabolism and their key transcriptional regulatory molecules PPARα (*Ppara*) and PGC-1α (*Ppargc1a*) in UUO samples were significantly lower than those in the sham-operated group (Figure 3B). The protein-protein interaction network and interaction analysis revealed that PGC-1α played a central role in regulating fatty acid metabolism. Notably, the transcription factor Twist1 was identified as the hub gene in a network with multiple genes involved in fatty acid metabolism and was directly associated with PGC-1α (Figure 3C). Gene concentration analysis confirmed the strong enrichment of FAO in different expression pathways (Figure 3D).

### Twist1 enhanced lipid accumulation via dysregulation of the FAO pathway in PTCs

**Figure 4:**
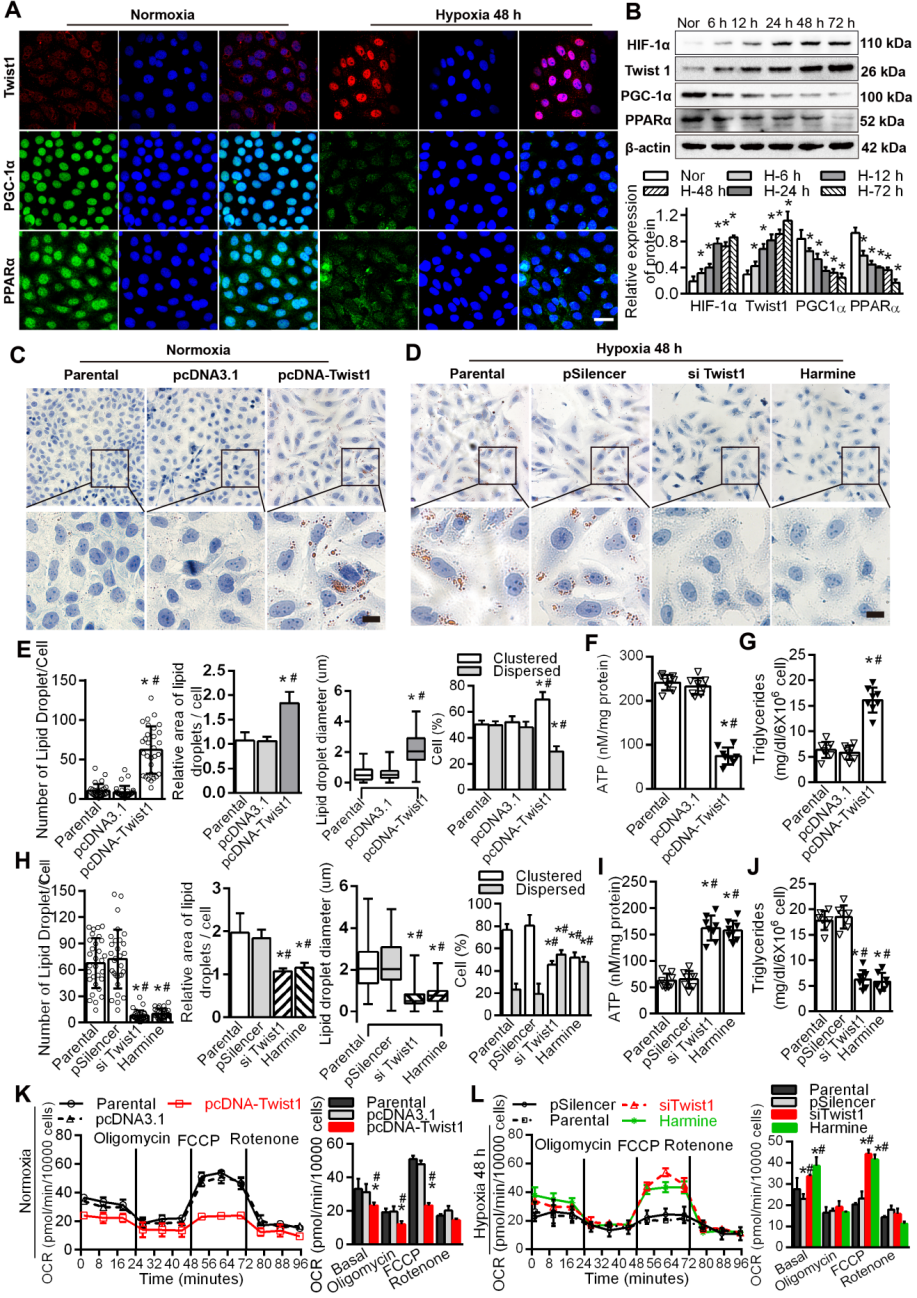
** Twist1 enhances lipid accumulation via derangement of the FAO pathway in PTCs. A.** Immunofluorescence analyses of Twist1, PGC-1α and PPARα expressions in the HK-2 normoxia and after hypoxia 48 h (green) with DIPA (delta-interacting protein A) in blue. Scale bar, 10 µm. **B.** The expression of HIF-1α, Twist1, PGC-1α and PPARα in HK-2 cells under normoxia (Nor) and hypoxia for 6 h, 12 h, 24 h, 48 h and 72 h detected by Western Blot. *P < 0.05 compared with the normoxia. **C and D.** Lipid droplets were detected by ORO staining in HK-2 cell transfected with Twist1 overexpression plasmid pcDNA-Twist1 or control plasmid pcDNA3.1 and control (Parental). Scale bar, 25 µm. **E and H.** The number, relative area, diameter, clustering and dispersion patterns of lipid droplets in HK-2 cells transfected with pcDNA-Twist1, pcDNA3.1, siTwist1 or Harmine was detected by HCS Studio Cell Analysis Software. **F and I.** The ATP content of HK-2 transfected with pcDNA-Twist1, pcDNA3.1, siTwist1 or Harmine was detected. **G and J.** Triglyceride of HK-2 transfected with pcDNA-Twist1, pcDNA3.1, pSilencer, siTwist1 or Harmine was detected. *P < 0.05 compared with the parental, ^#^*P* < 0.05, compared with the pcDNA3.1/pSilencer. **K.** OCR in HK-2 treated with pcDNA-Twist1 and pcDNA3.1 or not treated (parental). Representative traces are shown on the right, and summary data analyzed for 24 wells for three independent experiments are shown on the left. Where indicated, oligomycin (1 µM), FCCP (1 µmol/L) and rotenone (1 µmol/L) were added. **L.** OCR in HK-2 treated with siTwist1 or Harmine, as compared to parental or pSilencer. Representative traces are shown on the right, and summary data analyzed for 24 wells for three independent experiments are shown on the left. Where indicated, oligomycin (1 µM), FCCP (1 µmol/L) and rotenone (1 µmol/L) were added. *P < 0.05 compared with the parental, ^#^*P* < 0.05, compared with the pcDNA3.1/pSilencer throughout the figure by unpaired Student's t test. Each data point represents the mean ± SEM of 3 independent samples.

Immunofluorescence results showed that the expression of Twist1 was significantly increased in HK-2 cells cultured under hypoxic conditions for 48 h, mainly in the nucleus. The expression of PGC-1α and PPARα in HK-2 cells cultured under normoxic conditions was significantly higher than that in HK-2 cells cultured under hypoxia, mainly in the nucleus and a small amount in the cytoplasm (Figure 4A), with correspondingly higher levels of PGC-1α and PPARα (Figure 4B). The levels of hypoxia-inducible Factor 1α (HIF-1α) and Twist1 increased after exposure to hypoxia (^*^P < 0.05) (Figure 4B). Overexpression of Twist1 appeared to increase the number and relative size of lipid droplets in HK-2 cells, promote lipid droplet aggregation and reduce hydrolysis under normoxia (Figure 4C and E). Twist1 overexpression was also associated with decreased ATP synthesis and increased triglyceride content in HK-2 cells (Figure 4F-G).

Harmine, a specific inhibitor of Twist1, can inhibit the expression and result in the degradation of Twist1 20. Silencing Twist1 with the plasmid siTwist1 and Harmine under hypoxia reduced the number and relative size of the lipid droplets in HK-2 cells and reduced lipid droplet aggregation to promote hydrolysis (Figure 4D, H and Figure S4). ATP synthesis was also significantly increased, whereas triglyceride content was decreased in HK-2 cells (Figure 4I-J). Overexpressing Twist1 resulted in a decreased baseline OCR and decreased OCR induced by FCCP, indicating lower activity of fatty acid metabolism in PTCs under normoxia (Figure 4K). SiTwist1 and Harmine increased the baseline OCR and significantly promoted the OCR induced by FCCP under hypoxic conditions, indicating higher activity of fatty acid metabolism (Figure 4L).

### Twist1 promoted renal interstitial fibrosis by inhibiting PGC-1α and regulating fatty acid metabolism

**Figure 5:**
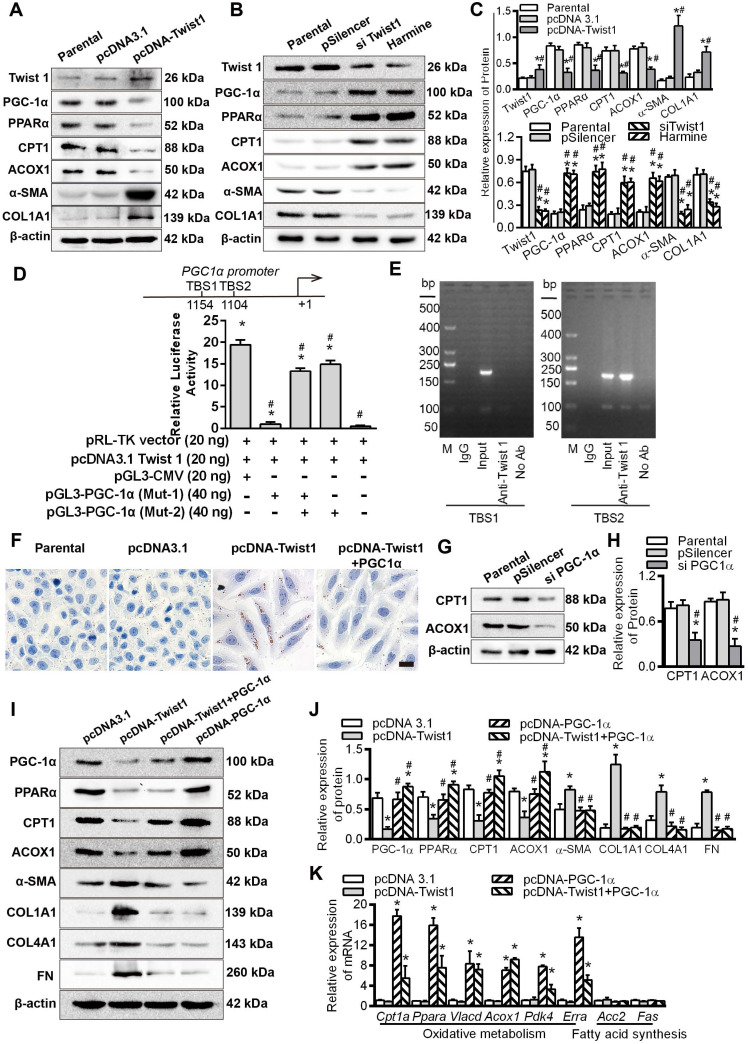
** Twist1 promoted renal interstitial fibrosis by inhibiting PGC-1α and regulating abnormal fatty acid metabolism. A and B.** The expression of Twist1, PGC-1α, PPARα, CPT1, ACOX1, α-SMA and COL1A1 in the HK-2 cells transfected with parental, pcDNA3.1, pcDNA-Twist1, pSilencer, siTwist1 or Harmine was detected by Western Blot. **C.** Histogram showing density correction for the loading control (β-actin). *P < 0.05 compared with parental, ^#^*P* < 0.05 compared with the pcDNA3.1 / pSilencer. D. Luciferase activity of the PGC-1α promoter reporter gene. HK-2 cells were transfected with 20 ng of reporter constructs and 1 mg of pcDNA3.1-Twist1 in combination with 0.2 ng of the pRL-TK vector and/or 40 ng of mutation vectors of PGC-1α promoter (Mut-1 and Mut-2) incubated for 24 h. The luciferase activities are reported as relative light units of firefly luciferase activity normalized to Renilla luciferase activity. HK2 cells were co-transfected with PGL3-CMV, pcDNA 3.1 Twist1 and pRL-TK vector as the positive control group, pRL-TK vector and pcDNA 3.1 Twist1 were co-transfected into HK-2 group as negative control group. *P < 0.05 compared with the negative control, *P < 0.05 compared with the positive control, n = 3 independent experiments. **E.** Chromatin immunoprecipitation (ChIP) analysis of Twist1 binding to the PGC-1α promoter in HK-2 cells under hypoxic conditions. The reaction controls included immunoprecipitation performed using a nonspecific IgG monoclonal antibody (Cont IP). PCR was performed using whole cell genomic DNA (input). The data are representative examples of 3 independent experiments. **F.** Lipid droplet accumulation was detected by ORO staining in the HK-2 cells transfected with pcDNA3.1, pcDNA-Twist1 and/or pcDNA-PGC-1α. Scale bar, 25 µm. **G.** The expression of CPT1 and ACOX1 in the HK-2 cells transfected with the control (parental), pSilencer and/or siPGC-1α was detected by Western Blots. **H.** Histogram showing density corrected for the loading control (β-actin). *P < 0.05 compared with the parental, ^#^*P* < 0.05 compared with the pSilencer. **I.** Expression of PGC-1α, PPARα, CPT1 and ACOX1 in HK-2 cells transfected with pcDNA3.1, pcDNA-Twist1 and/or pcDNA-PGC-1α detected by Western Blots. **J.** Histogram showing density corrected for the loading control (β-actin). *P < 0.05 compared with the pcDNA3.1, ^#^*P* < 0.05 compared with the pcDNA-Twist1. K. Quantitative RT-PCR results of mRNA expression of the fatty oxidative metabolism and acid synthesis-related genes in the HK-2 cells transfected with pcDNA3.1, pcDNA-Twist1 and/or pcDNA-PGC-1α under normoxia. *P < 0.05 compared with the pcDNA3.1. Each data point represents the mean ± SEM of 3 independent samples.

HK-2 cells cultured under normoxia were transfected with a Twist1 overexpression plasmid. Compared with those of the control group, the levels of the fibrosis factors α-SMA and COL1A1 were promoted, while the levels of PGC-1α and its downstream target genes PPARα, CPT1 and ACOX1 were significantly decreased (Figure 5A). Downregulation of Twist1 with siTwist1 and Harmine significantly decreased the expression of Twist1 and fibrosis factors and significantly alleviated the decrease in FAO-related PGC-1α, PPARα, CPT1 and ACOX1 proteins induced by hypoxia (Figure 5B). Histograms on the right show the relative protein expression by Western blot (Figure 5C).

To further reveal the transcriptional regulation of PGC-1α by Twist1, we identified 2 Twist1 binding sites (TBS) (CANNTG) using *in silico* analysis of the promoter region of PGC-1α 31, 32. The pGL3-CMV vector and Twist1 overexpression vector (pcDNA3.1 Twist1) and/or the mutant vectors of PGC-1α promoter (Mut-1, Mut-2) were co-transfected into HK-2 cells. The luciferase activity of PGC-1α promoter vector mutated the promoter vector (Mut-1) of the TBS1 binding site was decreased by -18.41 ± 0.71 times compared with positive control (HK2 cells were co-transfected with PGL3-CMV, pcDNA 3.1 Twist1 and pRL-TK vector as the positive control group) (*P* < 0.05). Furthermore, compared with the promoters transfected with mutated TBS2 sites (Mut-2) and positive control, the PGC-1α Mut-2 vector showed -4.454 ± 0.80 times in luciferase activity compare with the positive control (Figure 5D). It was suggested that Twist1 had a direct transcriptional inhibition effect on PGC-1α. These findings were further confirmed by chromatin immunoprecipitation analysis of nuclei from HK-2 cells. The analysis showed that the 204 bp band containing the second potential binding site TBS2 (-1109 to -1104 region) was positive. No band was observed at the other site or for the control (Figure 5E), suggesting that Twist1 and the PGC-1α promoters directly bind via the proximal TBS2 site, decreasing PGC-1α transcription. After confirming that overexpression of Twist1 inhibited the expression of PPARα in HK-2 cells under normoxia, and silencing Twist1 under hypoxia promoted the expression of PPARα, we assessed whether Twist1 directly transcriptively regulated PPARα. The results of the luciferase reporter gene assay showed that there was no significant difference in the relative activity of luciferase in HK-2 co-transfected with Twist1 overexpression vector and PPARα promoter compared with negative control, pRL-TK plasmid, pGL3-PPARα and pRL-TK. It appears that Twist1 has no direct transcriptional activation effect on PPARα (Figure S5A). Overexpression of PGC-1α increased the mRNA level of PPARα, whereas silencing PPARα suppressed PPARα mRNA levels (Figure S5B). Further luciferase reporter gene assays also confirmed the direct transcriptional activation of PPARα expression by PGC-1α (Figure S5C).

Twist1 regulates FAO through PGC-1α, overexpression of Twist1 in HK-2 cells under normoxia, promoting intracellular lipid droplet accumulation, increased triglyceride synthesis and decreased ATP. However, The combination of overexpression of Twist1 and PGC-1α significantly alleviated the increase in lipid droplet accumulation, the increase in triglyceride synthesis and the decrease in ATP synthesis caused by Twist1 alone (Figure 5F and Figure S5D-E). Silencing PGC-1α decreased CPT1 and ACOX1 (Figure 5G-H), whereas upregulation of PGC-1α increased the protein levels of the downstream target genes CPT1 and ACOX1 (Figure 5I-J). Co-overexpression of Twist1 and PGC-1α in HK-2 cells significantly alleviated the decrease in PGC-1α, PPARα, CPT1 and ACOX1 induced by upregulation of Twist1 under normoxia and significantly decreased the increase in α-SMA, COL1A1, COL4A1 and FN induced by upregulation of Twist1 (Figure 5I-J). However, overexpression of Twist1 alone had no effect on non-PGC-1α target genes, such as *Acc2* and *Fas*, which are involved in fatty acid synthesis (Figure 5K).

### Knockout of the Twist1 gene in renal tubules slowed renal fibrosis by restoring abnormal fatty acid metabolism

PEPCK-CRE transgenic mice carry a Cre recombinant gene controlled by a modified renal proximal tubule-specific PEPCK promoter that mainly drives Cre expression in renal PTCs 33, 34. We bred proximal tubule-specific Twist1 gene knockout mice with a PEPCK-Cre and Twist1 (flox/flox) transgenic modification (named PT-*Twist1*^-/-^) (Figure S1A-B) to explore the effects of Twist1 intervention on fatty acid metabolic recovery and inhibition of renal interstitial fibrosis *in vivo*.

**Figure 6:**
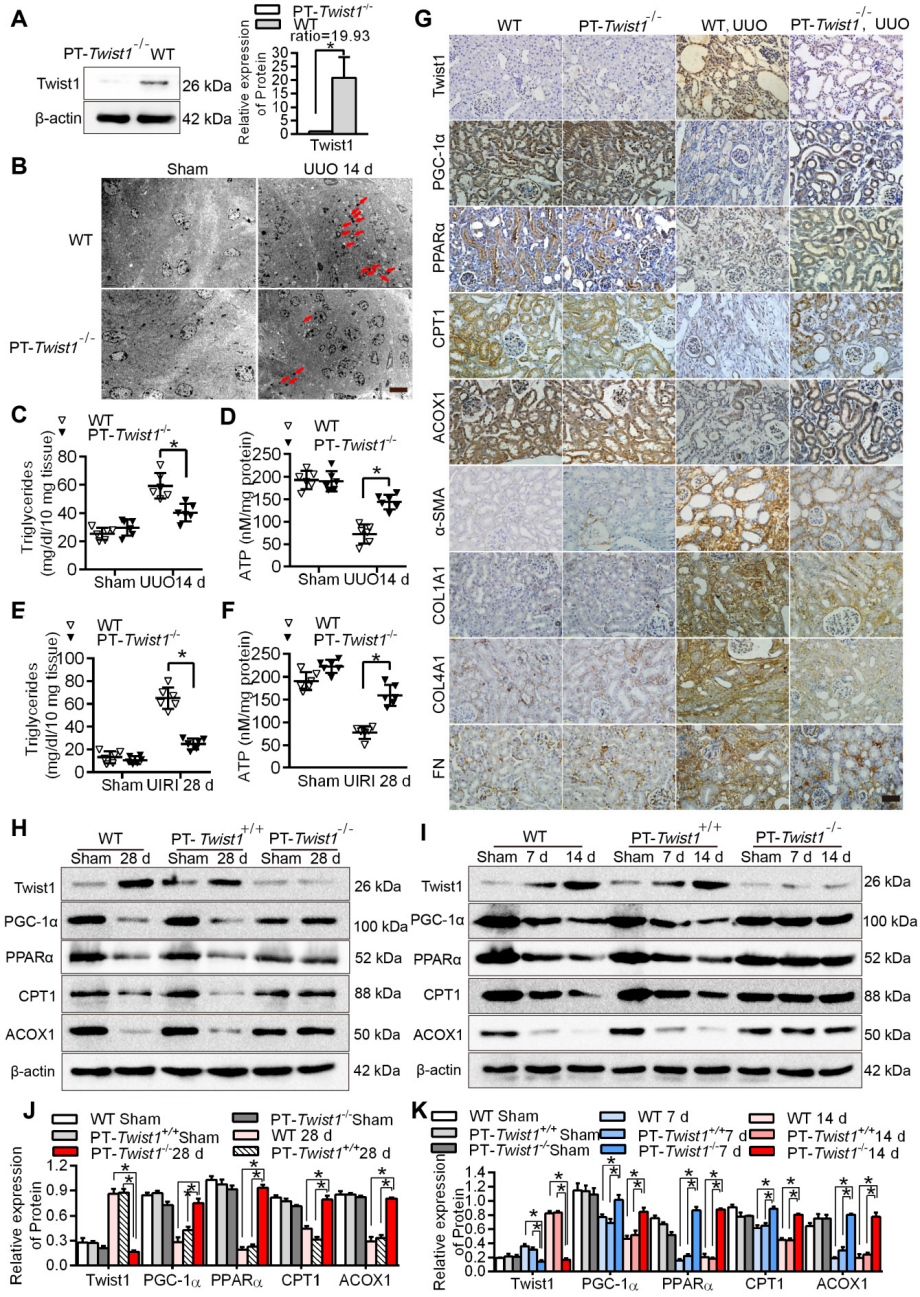
** Knockout of Twist1 gene in renal tubules slowed down renal fibrosis by restoring fatty acid metabolism disorders. A.** Western blot analyses showed that Twist1 expression was decreased significantly in the kidneys of PT-*Twist1*^-/-^ but not WT mice after UUO. **B.** Electron microscopic images showed lipid droplets (LDs) in the kidneys from the WT and PT-*Twist1*^-/-^ mice with UUO. Red arrows indicate lipid vacuoles. N indicates nucleus. Bar = 2 µm. **C.** Triglyceride levels were detected in the kidneys of the WT and PT-*Twist1*^-/-^ mice with UUO, *P < 0.05. **D.** The ATP level in the kidneys of the WT and PT-*Twist1*^-/-^ mice with UUO were detected, *P < 0.05. **E and F.** Triglyceride and ATP were detected in kidneys of the WT and PT-*Twist1*^-/-^ mice with UIRI, *P < 0.05. **G.** The expression of Twist1, PGC-1α, PPARα, CPT1, ACOX1, α-SMA, COL1A1, COL4A1 and FN was detected by immunohistochemical in the UUO-induced kidneys from the WT and PT-*Twist1*^-/-^ mice (400×). Bar = 50 µm. **H and I.** Twist1, PGC-1α, PPARα, CPT1 and ACOX1 protein expression in UIRI and UUO kidneys from WT, PT-*Twist1*^+/+^ and PT-*Twist1*^-/-^ mice. **J and K.** Histogram showing density correction for the loading control (β-actin). Data are presented as the mean ± SEM for each group of mice (n = 6 in the sham group; n = 6 in the UIRI group; n = 6 in the UUO group). *P < 0.05.

The protein level of Twist1 in the renal cortex of the PT-*Twist1*^-/-^ mice was significantly lower than that of the wild type mice (WT) (Figure 6A). The PT-*Twist1*^-/-^, littermate control PEPCK-Cre mice (named PT-*Twist1*^+/+^) and WT mice, were used to construct the UUO and UIRI models. On days 0 and 14 of UUO, and on days 0 and 28 of UIRI, the fibrotic kidneys were analyzed by H&E and Masson's trichrome staining (Figure S6A-B). Compared with WT and PT-*Twist1*^+/+^ UUO +14 days and UIRI +28 days renal tubule health status was significantly improved in PT-*Twist1*^-/-^mouse (Figure S6C-D), the positive rate of Masson's trichrome staining and the degree of renal interstitial fibrosis decreased significantly in kidneys of PT-*Twist1*^-/-^ mice (Figure S6E-H).

Further electron microscopic examination showed that lipid droplets were significantly lower in the PT-*Twist1*^-/-^ mice than the WT mice on the UUO + 14 days (Figure 6B). The content of triglyceride also significantly lower in kidney tissue of UUO model and UIRI model from PT-*Twist1*^-/-^ mice, while ATP was significantly higher (Figure 6C-F). The immunohistochemical results showed that compared with the UUO model in WT, the expression of Twist1 and fibrosis factor α-SMA, COL1A1, COL4A1 and FN was significantly lower, and the expression of PGC-1α, PPAR α, CPT1 and ACOX1 significantly higher on UUO + 14 days model in PT-*Twist1*^-/-^ mice (Figure 6G), with correspondingly the protein levels of Twist1 and fibrosis factor were diminished, and PGC-1α, PPARα, CPT1 and ACOX1 were ameliorated in the same models using PT-*Twist1*^-/-^ mice (Figure 6H-I). Figure 6J-K showed the results of protein quantitative analysis using Western blot.

### Novel Twist1 inhibitor Harmine prevented fatty acid metabolic disorders and fibrogenesis *in vivo*

**Figure 7:**
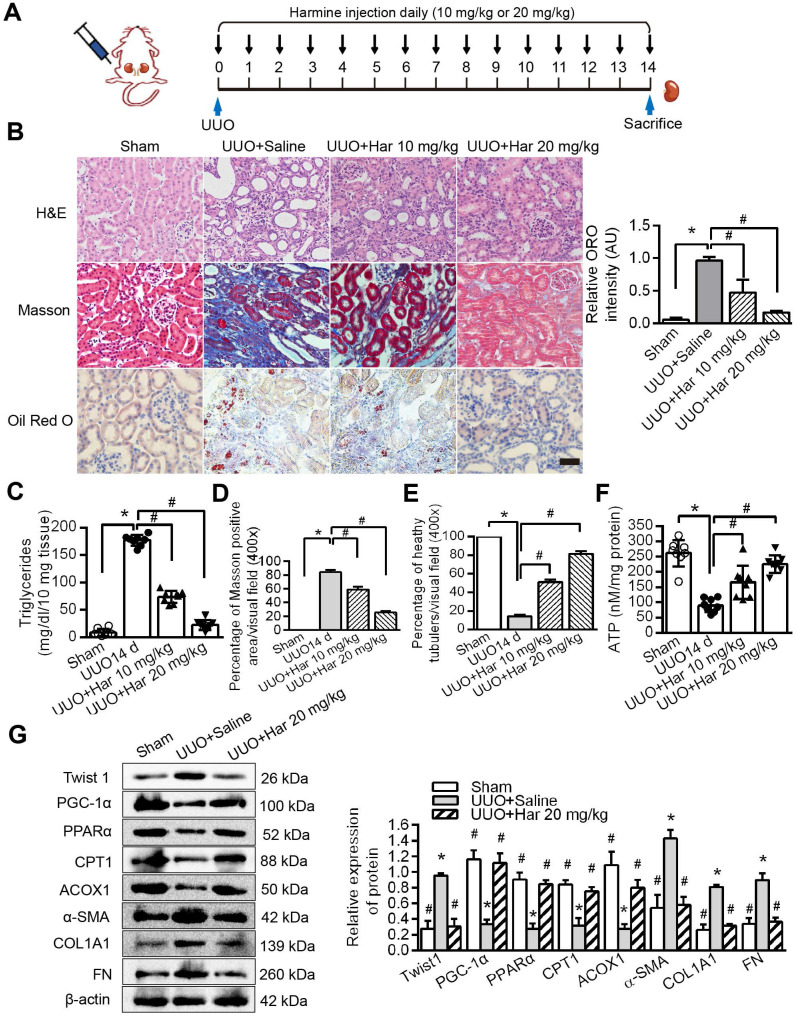
** Harmine prevented fatty acid metabolic disorders and fibrogenesis *in vivo*. A.** Schematic diagrams of the experimental procedure. **B.** Lipid accumulation analyzed by ORO staining and tubulointerstitial health and fibrosis by H&E and Masson trichrome staining. Right panels indicate the results of computer-assisted densitometric image analysis. Harmine ameliorated lipid droplet formation. Bar = 50 µm. Fibrosis **C and D** Triglycerides **E** ATP **F** in kidney tissue with UUO injury. Bar = 20 µm. ^*^P < 0.05, ^#^ *P* < 0.05. **G.** Western blot analysis showed decreased Twist1, α-SMA, COL1A1 and FN and increased PGC-1α, PPARα, CPT1 and ACOX1 expression in the UUO kidneys of the Harmine group. β-actin serves as an internal control. Data are presented as the mean ± SEM for each group of mice (n = 6 in each group). ^*^P < 0.05 compared with the Sham, ^#^ *P* < 0.05 compared with the UUO + saline group.

We suppressed Twist1 by intraperitoneal injection of Harmine in mice and then performed the UUO procedure (Figure 7A). Furthermore, Harmine treatment resolved the aggregation of lipid droplets and the increase in triglycerides caused by UUO (Figure 7B, tested by ORO and Figure 7C). These changes resulted in reduced renal fibrosis and increased health status and ATP (Figure 7D-F), as shown using Masson's trichrome and H&E staining. The protein levels of Twist1 and fibrosis factors were decreased significantly by Harmine in mouse kidneys with UUO, whereas the protein levels of PGC-1α, PPARα, CPT1 and ACOX1 were significantly higher (Figure 7G). Taken together, these results provide *in vivo* data showing that Twist1 plays a key role in blocking FAO and promoting TIF in mice with UUO.

## Discussion

Lipid accumulation in renal PTCs has received widespread attention for its potential role in acute renal injury, diabetic nephropathy, glomerular and tubulointerstitial diseases and other pathologies 29, 35-37. Renal TECs, especially PTCs, are filled with mitochondria and depend on oxidative phosphorylation for their energy needs. PTCs mainly rely on the oxidative energy supply of β-fatty acids under normoxic conditions in mitochondria 37. Biological stress (such as transient hypoxia) results in stagnation of FAO for a period of time, during which the metabolism of surviving PTCs is limited, inducing phenotypic transition to a low-level interstitial cell phenotype (i.e., EMT), with cytoskeleton rearrangement and production of extracellular matrix protein 1. Therefore, hypoxia-induced FAO blockade is a pivotal cause of acute kidney injury. Delayed recovery of FAO causes prolonged energy deficiency in PTCs, which explains why a short period of acute injury increases the risk of CKD 1, 38, 39. The accumulation of lipids caused by FAO disorders may also indirectly contribute to the promotion of inflammation and fibrosis 6, 40, 41. *Kang et al* found that disordered FAO in PTC may promote the development of TIF, which was related to lipotoxicity caused by lipid accumulation, resulting in an increase in the production of reactive oxygen species, a decrease in ATP, an increase in apoptosis, and an increase in the production of inflammatory cytokines and extracellular matrix 1. Improving FAO in PTCs can alleviate renal injury 42. Clinical evidence has shown that lipid droplets accumulate in TECs of patients with CKD 1, 43, and an increase in renal tubular lipid droplet accumulation is induced by a high-fat diet in mice 44, which also supports the hypothesis that damage to PTCs is related to changes in lipid metabolism. Therefore, defective lipid metabolism in PTCs is considered to be closely related to PTC dysfunction and ultimately to accelerated TIF. However, the molecular mechanism underlying the relationship between defective lipid metabolism and TIF caused by chronic hypoxia is unclear.

Twist1 is the key molecule that drives phenotypic changes in epithelial-mesenchymal transdifferentiation (i.e., EMT) in renal fibrosis and various tumors. In our previous study, we found that hypoxia-induced overexpression of Twist1 in PTCs promoted the progression of TIF by inducing EMT in PTCs. Furthermore, high expression of Twist1 was found in the renal tissue and serum of patients with CKD 14, 26, 45, 46. Knocking out Twist1 in mouse brown adipocytes can induce the expression of genes involved in oxidative metabolism and FAO, such as UCP-1 and CPT1 47, 48. Conversely, overexpression of Twist1 in C2C12 muscle tubules reduces PGC-1α-induced FAO 18. Twist1, which was overexpressed in brown adipose tissue, inhibited the target genes (CPT1β, UCP1 and ERRα) of PGC-1α and played a central role in regulating cellular energy metabolism by interacting with a variety of transcription factors (including PPARγ, PPARα, ERRα).

It has been reported that PGC-1α transgenic mice can effectively resist renal interstitial fibrosis 2. In this study, Twist1, a negative upstream regulator of PGC-1α, was found for the first time in the kidney, suggesting a tissue type-dependent function of the Twist1-PGC-1α-PPARα axis. This study showed that hypoxia-induced Twist1 activation and transcriptional inhibition of PGC-1α expression resulted in decreased FAO efficiency in TECs and subsequent TIF. Moreover, under normoxic conditions, overexpression of Twist1 significantly inhibited FAO in PTCs, and the cells developed a transdifferentiation phenotype (Figure 4 and Figure 5F), releasing more fibrosis factors and extracellular matrix. Treatment with the Twist1 inhibitor Harmine reduced these effects, suggesting that Twist1 can promote TIF induced by abnormal lipid metabolism (lipid accumulation). We detected that mice with conditional deletion of Twist1 in proximal PTCs (PT-*Twist1*^-/-^) were resistant to renal fibrosis. Inhibition of Twist1 restored FAO, suppressed extracellular matrix accumulation, and attenuated fibrosis, informing potential antifibrosis therapies.

We achieved effective results using Harmine to reduce Twist1 protein and alleviate disorders of FAO and its downstream effects in the kidney. However, mice treated with Harmine *in vivo* showed excitement and hyperactivity symptoms compared with the saline group, and a slight but not statistically significant weight loss (as shown in Table S7). We speculate that Harmine may act on the nervous system. Before Harmine is tested in clinical trials, further study of its wider effects is needed, and modifications to remove the risk of such effects may be needed. Our group is currently investigating the utility of Harmine treatment in the prevention of TIF.

**Figure 8:**
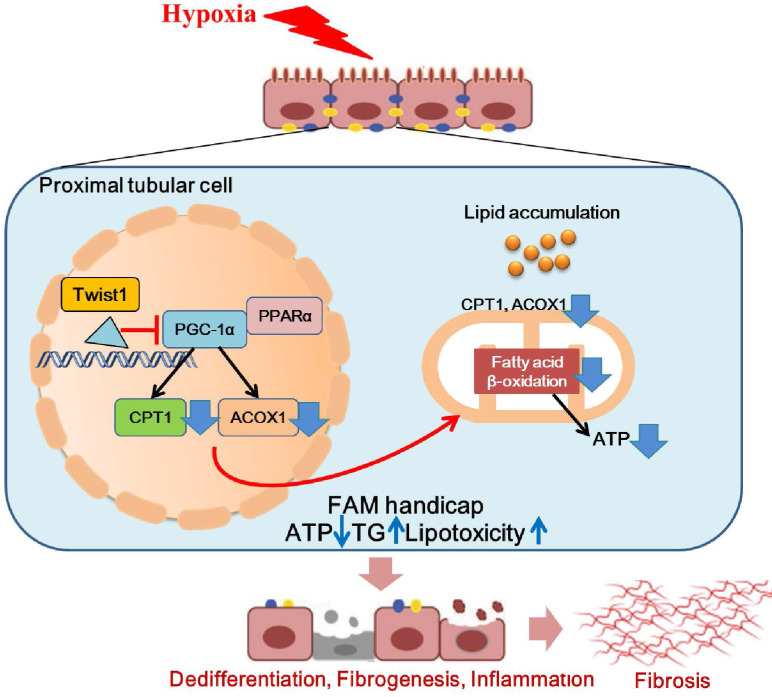
** Hypoxia-induced Twist1-PGC-1α axis mediated mitochondrial lipid metabolism contributes to progressive renal interstitial fibrosis.** We found that Twist1 increased under chronic hypoxia and pathogenic conditions, leading to mitochondrial dysfunction and intracellular lipid accumulation and defective FAO by inhibiting PGC-1α and the downstream target genes PPARα, CPT1 and ACOX1. This, in turn, leads to ATP depletion and triglyceride overload and consequently facilitates lipotoxicity-induced TIF.

In conclusion, we found that Twist1 increased under chronic hypoxia and pathogenic conditions, leading to mitochondrial dysfunction and intracellular lipid accumulation and defective FAO by inhibiting PGC-1α and the downstream target genes PPARα, CPT1 and ACOX1. This, in turn, leads to ATP depletion and triglyceride overload and consequently facilitates lipotoxicity-induced TIF (Figure 8). Blocking the expression of Twist1 can partially alleviate the development of hypoxia-mediated TIF. Therefore, we propose that blocking the abnormalization of Twist1 may be a promising treatment to prevent the progression of CKD and the subsequent development of or progression to TIF.

## Supplementary Material

Supplementary figures and tables.

## Abbreviations

FAO: fatty acid oxidation
PTCs: proximal tubular cells
TIF: renal tubulointerstitial fibrosis
TECs: tubular epithelial cells
ROS: reactive oxygen species
b-HLH: basic helix-loop-helix
EMT: epithelial-mesenchymal transition
UUO: unilateral ureteral obstruction
UIRI: unilateral ischemia reperfusion injury
PPARα: peroxisome proliferator-activated receptor alpha
ACOX1: peroxisomal acyl-coenzyme A oxidase 1
OCR: oxygen consumption rate
ORO: Oil Red O
COL1A1: Collagen I
COL4A1: Collagen IV
FN: Fibronectin
PT-*Twist1*^-/-^: mice with conditional deletion of Twist1 in proximal TECs
PT-*Twist1*^+/+^: littermate control PEPCK-Cre mice
