# Twist1 downregulation of PGC-1α decreases fatty acid oxidation in tubular epithelial cells, leading to kidney fibrosis: Erratum

**DOI:** 10.7150/thno.82539

**Published:** 2023-02-22

**Authors:** Limin Liu, Xiaoxuan Ning, Lei Wei, Ying Zhou, Lijuan Zhao, Feng Ma, Ming Bai, Xiaoxia Yang, Di Wang, Shiren Sun

**Affiliations:** 1 Department of Nephrology, Xijing Hospital, Fourth Military Medical University, No. 127 Chang le West Road, Xi'an, Shaanxi, 710032, China.; 2 School of Medicine, Northwest University, 229 Taibai North Road, Xi'an, Shaanxi, 710069, China.; 3 Department of Geriatrics, Xijing Hospital, Fourth Military Medical University, No. 127 Chang le West Road, Xi'an, Shaanxi, 710032, China.

The authors regret that the original version of our paper, unfortunately, contained two incorrect pictures in Figure 2A, where the images for the H&E and Masson staining pictures of the uIRI Sham were incorrect. The correct version of Figure 2A is shown below.

The correction made in this erratum does not affect the original data and conclusions. The authors apologize for any inconvenience that the errors may have caused.

## Figures and Tables

**Figure 2:**
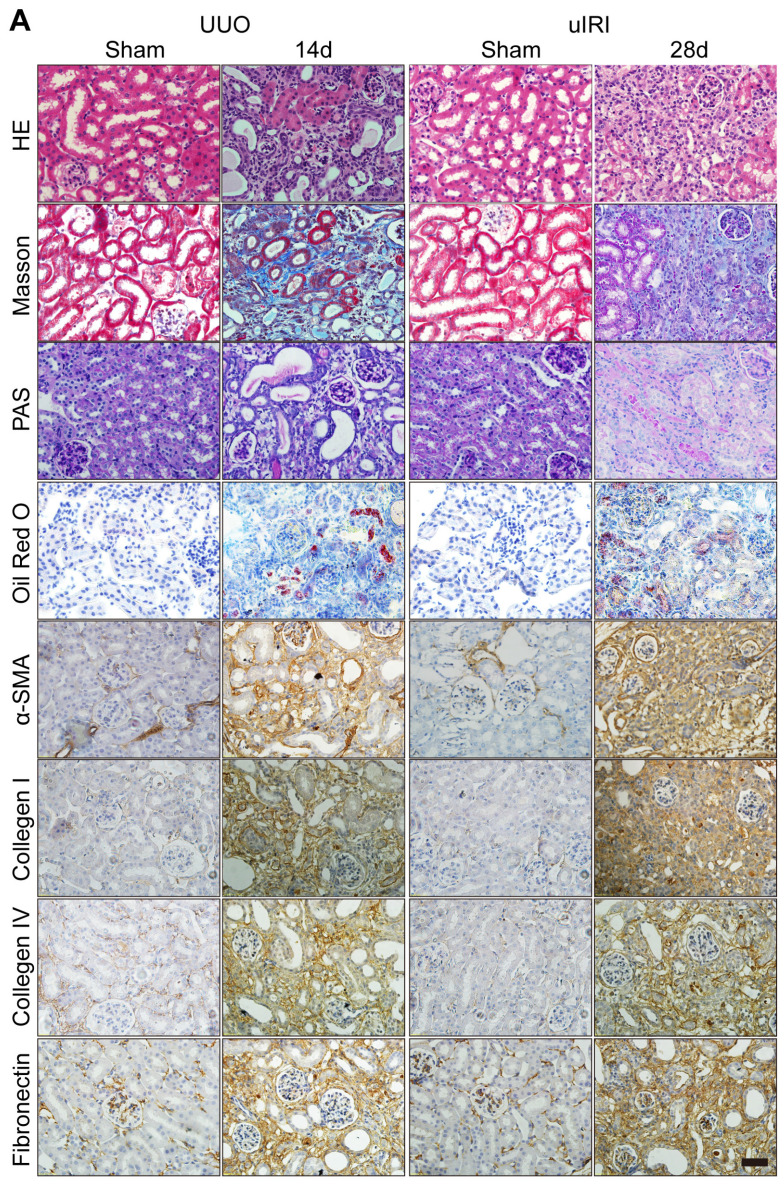
Corrected figure for original Figure 2A.

