# Supplementary material for: Twist1 downregulation of PGC-1α decreases fatty acid oxidation in tubular epithelial cells, leading to kidney fibrosis
Source: Theranostics. 2022 May 1;12(8):3758–75. doi: 10.7150/thno.71722 (PMC9131259; doi:10.7150/thno.71722)

Supplemental information

Twist1 downregulation of PGC-1α decreases fatty acid oxidation in tubular epithelial cells, leading to kidney fibrosis

Limin Liu, Xiaoxuan Ning, Lei Wei , Ying Zhou, Lijuan Zhao, Feng Ma, Ming Bai, Xiaoxia Yang, Di Wang, Shiren Sun

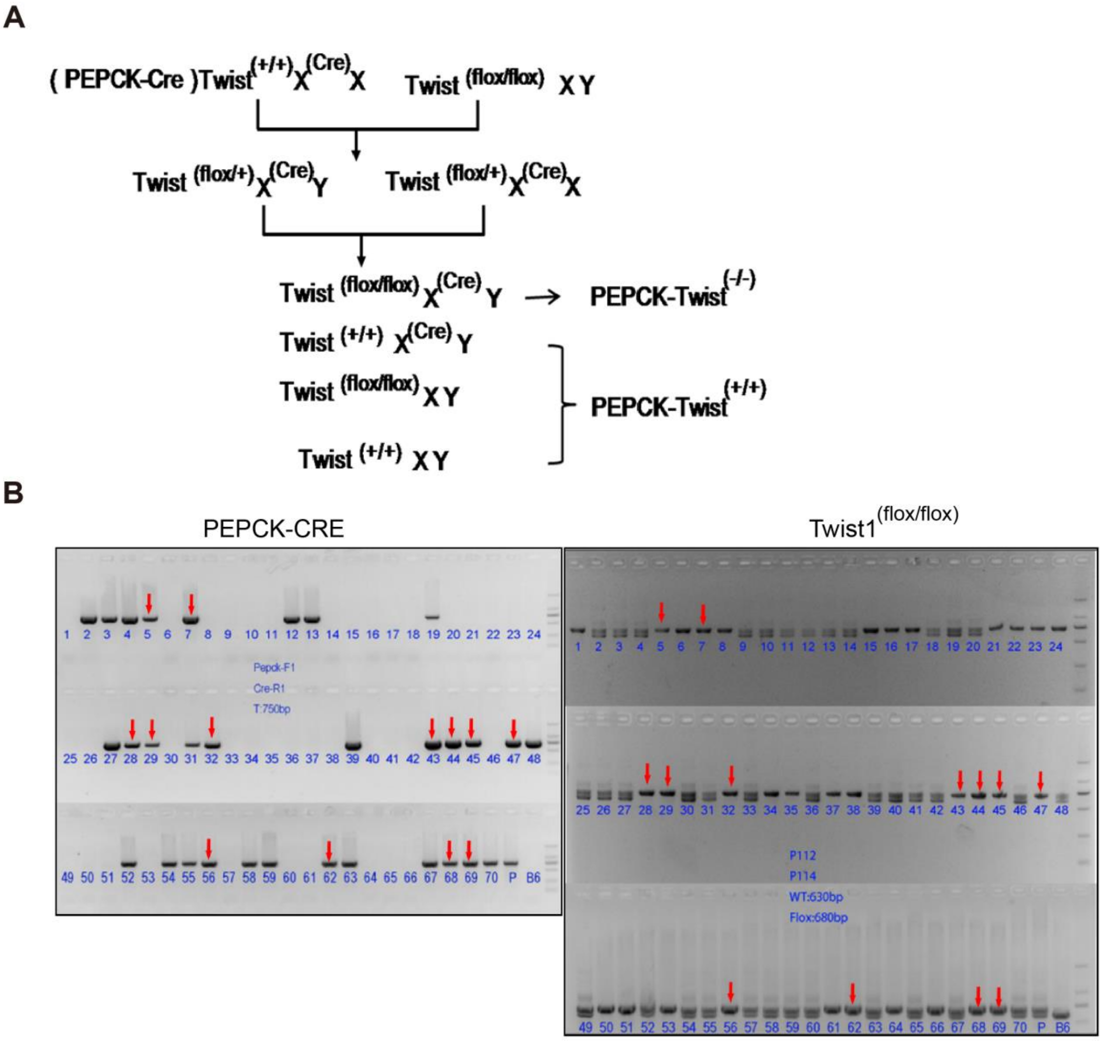

Figure S1 Mouse model of targeted deletion of Twist1 from renal proximal tubules.

Related to Figure 6.

(A) Breeding protocol for the production of proximal tubule-specific Twist1 knockout (PT-*Twist1*$^{-/-}$) mice. This study uses only male littermate mice with confirmed genotypes for experiment. **(B)** Genomic DNA was isolated from renal cortex of PT-*Twist1*$^{-/-}$ and PT-*Twist1*$^{+/+}$ mice. PCR genotyping was performed to confirm the deletion of Twist1 from the renal cortex. DNA samples (50 ng per lane) were amplified by PCR using primers for PEPCK-Cre and Twist1 (flox/flox) (the sequence shown in the concise method section). Actin was also amplified as control. The same number represents the same mice, and the glue positive strip on the left shows PEPCK-CRE， 680 bp on the right side represents Twist1 (flox/flox), 630 bp stands for Twist1 (+/+).

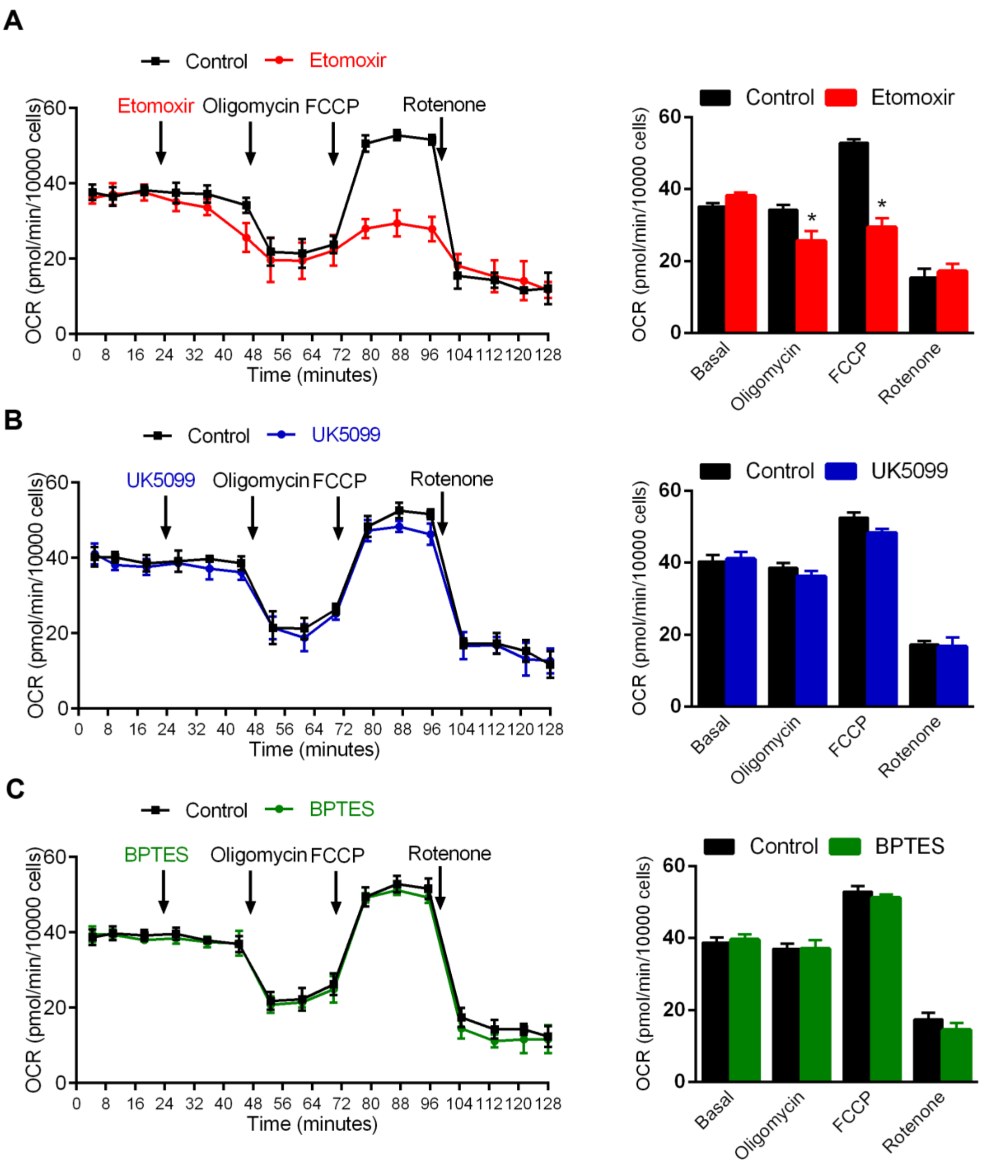

Figure S2 Mitochondrial substrate requirements or dependence of PTCs cells.

Related to Figure 1 and 4.

A-C.The oxygen consumption rate (OCR) in HK-2 cells was measured using a Seahorse XF flux analyzer. long-chain fatty acid [LCFA] oxidation was inhibited by etomoxir (4 μmol/L), glucose/pyruvate was antagonized by UK5099 (2 μmol/L), glutamate synthesis was suppressed by Bis-2-(5-phenylacetamido-1,3,4-thiadiazol-2-yl)ethylsulfide (BPTES) (3 μmol/L). Parameters of substrate oxidation pressure test for HK-2 cells: basal respiration, acute response, and maximum respiration. Representative traces are shown on the left, and the summary data analyzed for 24 wells from 3 independent experiments are shown on the right. Where indicated, oligomycin (1 μM), FCCP (1 μmol/L) and rotenone (1 μmol/L) were added. Measurements were performed in DMEM supplemented with 10 mmol/L glucose, 1 mmol/L pyruvate and 2 mmol/L glutamine, pH = 7.4. The control group was without inhibitor. Data are mean values ±SEM, n = 3, $^{*}P < 0.05$.

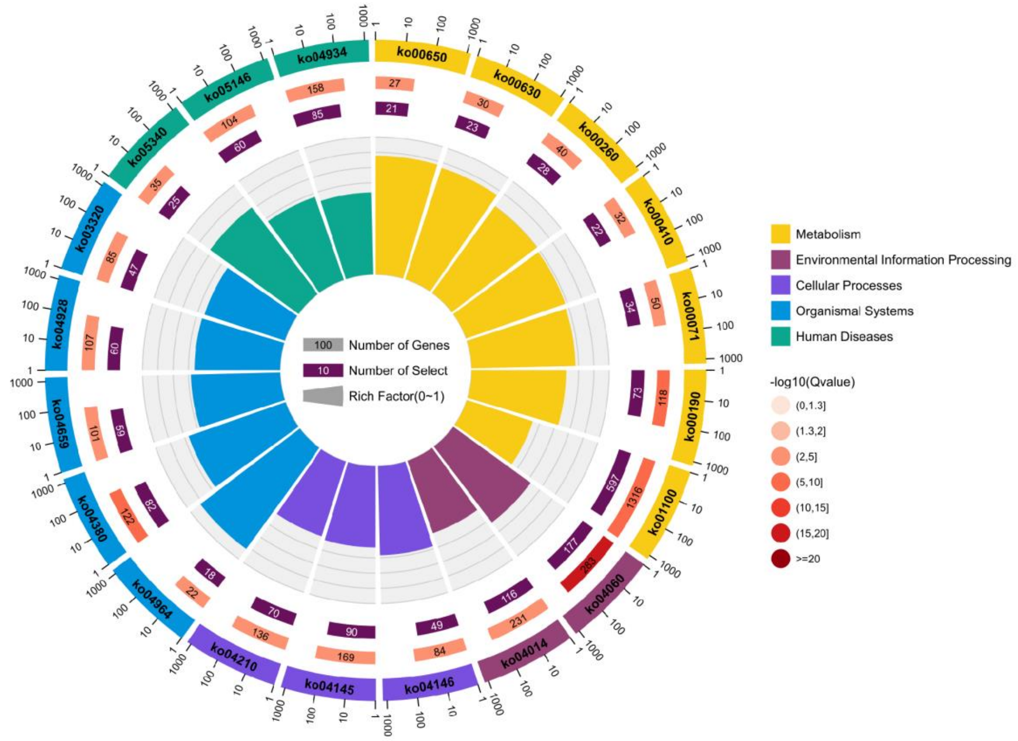

KO ID	Description
KO:04060	Environmental Information Processing
KO:01100	Metabolism
KO:04380	Development
KO:00190	Energy metabolism
KO:00071	Lipid metabolism
KO:04659	Immune system
KO:04145	Transport and catabolism
KO:00630	Carbohydrate metabolism
KO:04934	Endocrine and metabolic diseases
KO:05146	Infectious diseases
KO:00650	Carbohydrate metabolism
KO:04964	Excretory system
KO:00260	Amino acid metabolism
KO:04014	Signal transduction
KO:05340	Immune diseases
KO:04928	Endocrine system
KO:04146	Transport and catabolism
KO:00410	Metabolism of other amino acids
KO:04210	Cell growth and death
KO:03320	Endocrine system

Figure S3 GO term analysis of upregulated pathways in UUO was performed.

Related to Figure 3.

UUO mice renal cortices were cut with laser capture microdissection (Thermo Scientific) to obtain renal tubules. GO term analysis of upregulated pathways in UUO was performed. The first layer indicates top 20 GO term and the number of the genes is shown in the outer layer. The second layer indicates the number of the genes in the

genome background and Q values for enrichment of the upregulated genes for the specified biological process. The third layer indicates the ratio of the upregulated genes and downregulated genes. The inner layer indicates the enrichment factor of each GO term. GO, Gene Ontology (top). Description of the GO term (bottom).

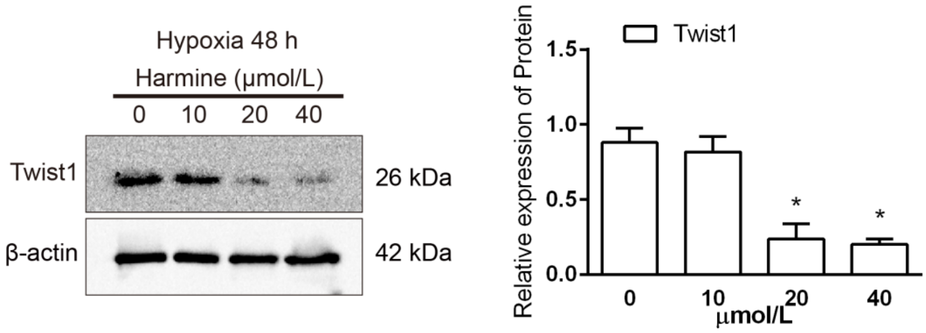

Figure S4 Harmine treatment leaded to Twist1 protein degradation.

Related to Figure 4.

Left, representative Western blots demonstrating that 48 h of Harmine treatment promotes Twist1 degradation in a dose-dependent manner in hypoxia treated 48 h HK-2 cell line; right, Relative quantitative protein analysis of Twist1. $^{*}P < 0.05$ compared with the 0 μmol/L.

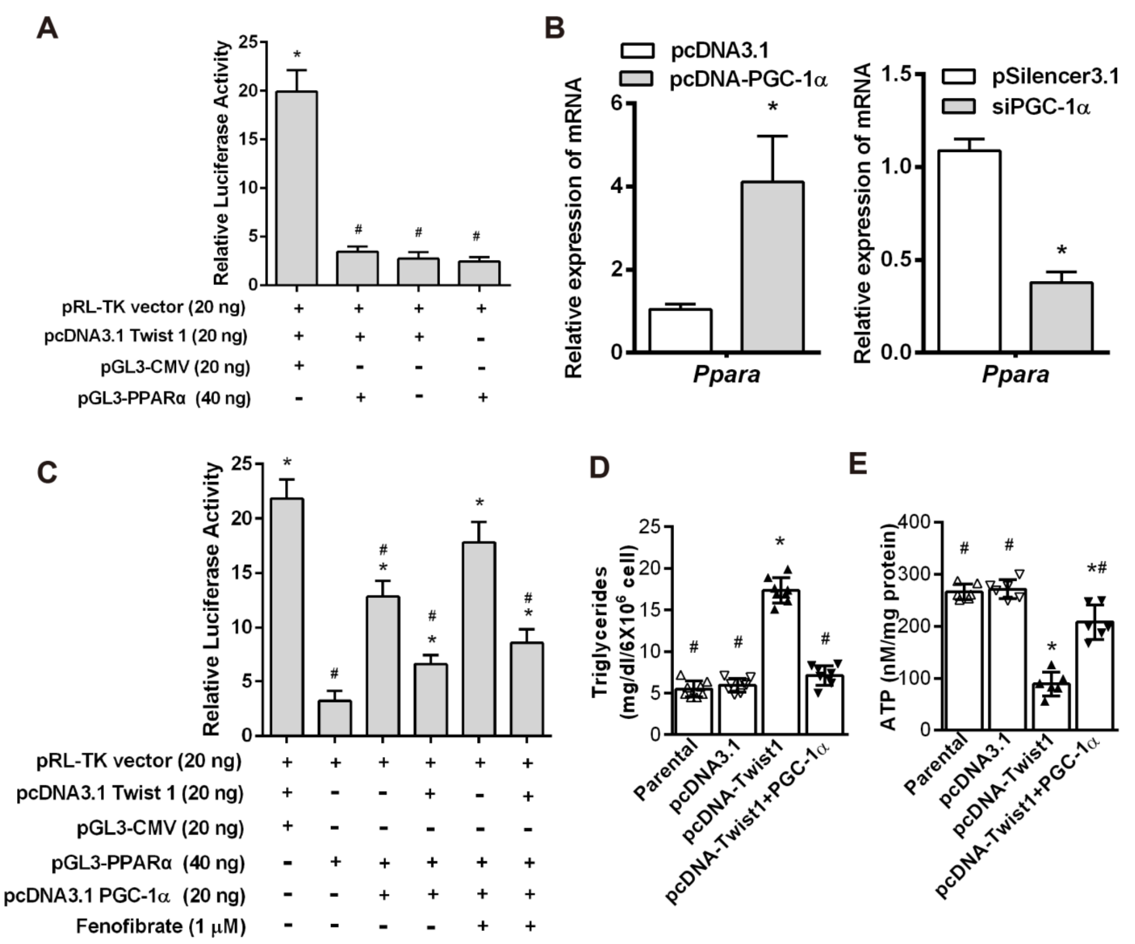

Figure S5 Twist1 has no direct transcriptional activation effect on PPARα, and PGC-1α directly transcriptively regulated PPARα.

Related to Figure 5.

(A) The relative luciferase activity of PPARα promoter reporter gene was detected. HK-2 cells were transfected with 40 ng PPAR α promoter reporter gene plasmid pGL3-PPAR and/or 20ng pcDNA3.1-Twist1 and 20ng pRL-TK for 48 h. HK2 cells were co-transfected with pGL3-CMV, Twist1 overexpression plasmid and pRL-TK plasmid in the positive control group, while HK2 cells were co-transfected with Twist1 overexpression plasmid and pRL-TK plasmid as negative control group. The experiment was repeated 3 times to calculate the average value. $^{*}P < 0.05$ compared with the negative control, $^{\#}P < 0.05$ compared with the positive control. **(B)** Quantitative RT-PCR analysis of *Ppara* following 48 h of transfection of pcDNA3.1-PGC-1α, pcDNA3.1, pSilencer or si PGC-1α. All plasmid treatment groups are normalized to untreated group. Data, mean ± SEM (n = 3 technical replicates). **(C)** HK-2 cells were transfected with 40 ng PPARα promoter and 20 ng pcDNA3.1-Twist1, 20 ng pcDNA3.1-PGC-1α or PPARα agonist Fenofibrate 1 μM for 48 h. The relative value of luciferase activity relative to renilla luciferase activity was analyzed. HK2 cells were co-transfected with pGL3-CMV,

pcDNA3.1 Twist1 and pRL-TK as the positive control group, and HK2 cells were co-transfected with pGL3-PPAR α and pRL-TK plasmid as the negative control group. The experiment was repeated 3 times to calculate the average value. $^{*}P < 0.05$ compared with the negative control, $^{\#}P < 0.05$ compared with the positive control. **(D)** Triglyceride of HK-2 transfected with pcDNA3.1, pcDNA-Twist1 and/or pcDNA-PGC-1α was detected. $^{*}P < 0.05$ compared with the parental, $^{\#}P < 0.05$ compared with the pcDNA-Twist1. **(E)** ATP of HK-2 transfected with pcDNA3.1, pcDNA-Twist1 and/or pcDNA-PGC-1α was detected. $^{*}P < 0.05$ compared with the parental $^{\#}P < 0.05$ compared with the pcDNA-Twist1.

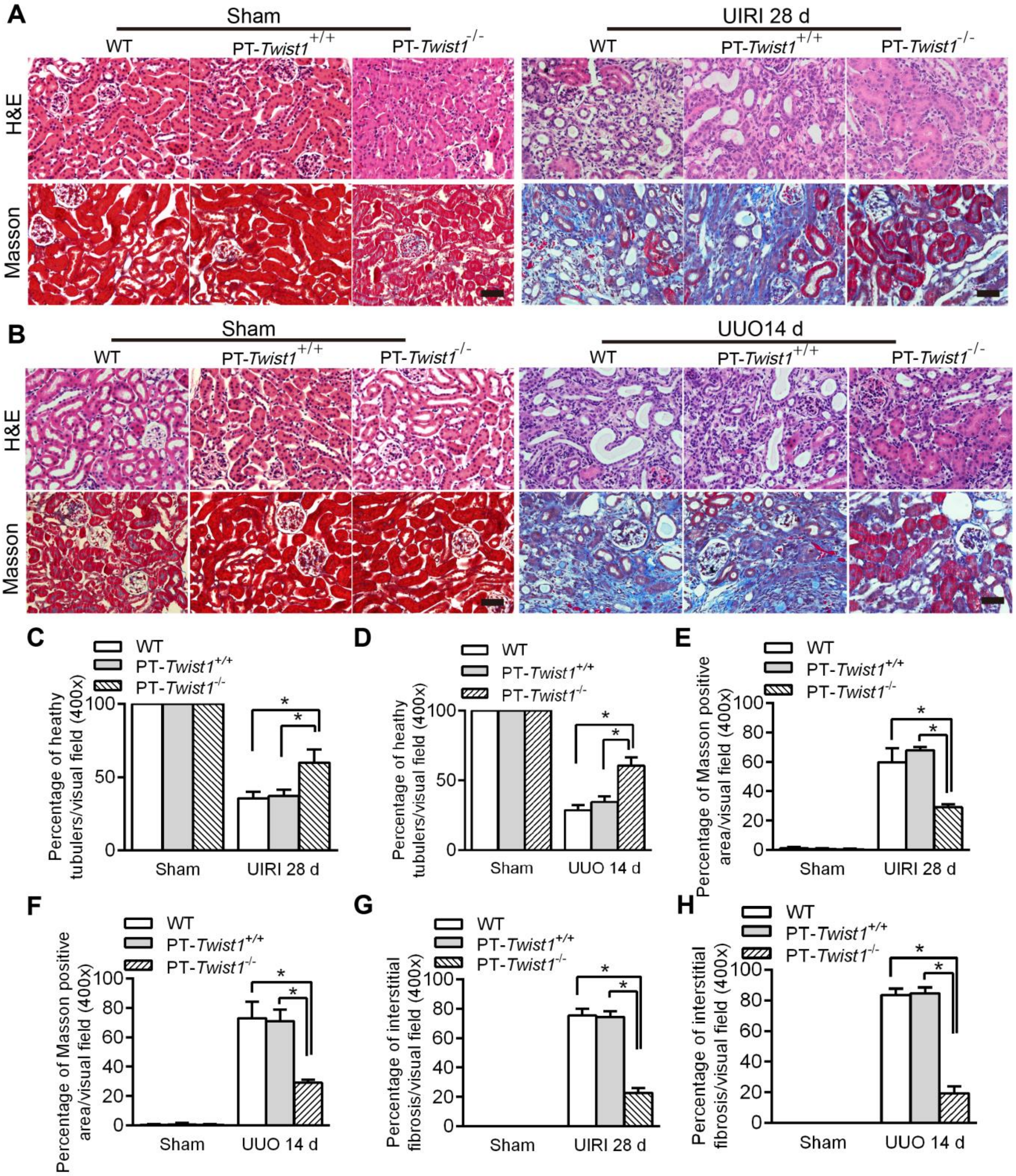

Figure S6 Construction of UIRI and UUO models with PT-*Twist1*$^{-/-}$ mice.

Related to Figure 6.

(A) UUO models were built with WT, PT-*Twist1*$^{+/+}$ and PT-*Twist1*$^{-/-}$ mice with kidneys harvested on day 14, **(B)** UIRI models were built for the same groups with kidneys harvested on day 28. H&E and Masson were used to detect pathological and fibrotic changes in kidney tissue in each group, bar = 20 μm. Renal tubule health status was analyzed in WT, PT-*Twist1*$^{+/+}$ and PT-*Twist1*$^{-/-}$ mice with UIRI **(C)** and UUO **(D)**. Renal tubule health status

was analyzed in WT, PT-*Twist1*$^{+/+}$ and PT-*Twist1*$^{-/-}$ mice with UIRI **(C)** and UUO **(D)**. The positive rate of Masson was analyzed in WT, PT-*Twist1*$^{+/+}$ and PT-*Twist1*$^{-/-}$ mice with UIRI **(E)** and UUO **(F)**. **(G) (H)** Percentage of interstitial fibrosis of WT, PT-*Twist1*$^{+/+}$ and PT-*Twist1*$^{-/-}$ mice with UIRI and UUO. $^{*}P < 0.05$ throughout the figure by unpaired Student's *t* test. Each data point represents the mean ± SEM of 8 mice for each group.

Table S1 Gene full name and sequences of primers used in this study.

Related to Figure 5 and Figure S4.

Gene symbol	Gene full name	Primers used for real-time QPCR
Human *Cpt1*	Carnitine palmitoyltransferase 1	F: 5'-GGATACAGAAGTGAAGACCCGGATA-3'<br>R: 5'-TTTGAAGACAACAAACGTGAACGAC-3'
Human *Ppara*	Peroxisome proliferative activated receptor alpha	F: 5'-TCCTGAGCCATGCAGAATTTAC-3'<br>R: 5'-AGTCTAAGGCCTCGCTGGTG-3'
Human *Vlcad*	Acyl-coenzyme A dehydrogenase, very long chain	F: 5'-GCCAGGGCAGAATCGAAGT-3'<br>R: 5'-TGGTAAGCTGGCCTTTGAACAT-3'
Human *Acox1*	Acyl Coenzyme A oxidase	F: 5'-TCTTGGAGATCCCTGTGAATCAATA-3'<br>R: 5'-GCAGCTACTGCTTGAAGACAACCTA-3'
Human *Pdk4*	Pyruvate dehydrogenase kinase, isoenzyme 4	F: 5'-GCAGTAGTCCAAGATGCCTTTGA-3'<br>R: 5'-AATACTGGTCGCAGAGCATCTTT-3'
Human *Erra*	Estrogen-related receptor alpha	F: 5'-AGCAAGCCCCGATGGA-3'<br>R: 5'-GAGAGGCCTGGGATGCTCTT-3'
Human *Acc2*	Acetyl-coenzyme A carboxylase beta	F: 5'-CCTACTATGAGGCCCAGCATGT-3'<br>R: 5'-TCGGCCTCTCTTCACCAGAT-3'
Human *Fas*	Fatty acid synthase	F: 5'-TGATGATTCAGGGAGTGGATATTG-3'<br>R: 5'-CCGAGCCAGGGACTTCTTAGT-3'
Human *β-actin*	β-non-muscle	F: 5'-TGGCACCCAGCACAATGAA-3'<br>R: 5'-CTAAGTCATAGTCCGCCTAGAAGCA-3'

Table S2. Plasmids construction of Twist1.

Related to Figure 4, 5 and Figure S 4.

Gene name	Forward (5'to 3')	Reverse (5'to 3')
pcDNA-Twist1	5'- TACCGGACTCAGATCTCGAGCGCCACC ATGATGCAGGACGTGTCCAGCTC -3'	5'- TACCGTCGACTGCAGAATTCGTG GGACGCGGACATGGACCAG -3'

Table S3. Primer sequence of PGC1 promoter region.

Related to Figure 5 and Figure S4.

Gene name	Forward (5'to 3')	Reverse (5'to 3')
Promoter of PGC1	F: 5'-CTAGCTAGCTGATGGACACAAAGCAGCCT -3'	R: 5'-CCGCTCGAGAGTGCTGAGAATTCGCCCAA -3'

Table S4. Sequences of CMV vector

Related to Figure 5 and Figure S4.

Gene name	CMV-NheI-F<br>Forward (5'to 3')	CMV-XhoI-R<br>Reverse (5'to 3')
CMV	5'-CTAGCTAGCgttgacattgattattgactagtt-3'	5'-CCGCTCGAGgagctctgcttatatagacctcc-3'

Table S5. Sequences of Twist1 shRNA.

Related to Figure 4 and 5.

ID	5’	stem	loop	stem	3’
TWIST1-RNAi(2691-1)-a	GATCCC	tcCGCAGTCTTACGAGGAGCT	CTCGAG	AGCTCCTCGTAAGACTGCGGA	TTTTTGGAT
TWIST1-RNAi(2691-1)-b	AGCTATCCAAAAA	tcCGCAGTCTTACGAGGAGCT	CTCGAG	AGCTCCTCGTAAGACTGCGGA	GG

Table S6 Demographic and clinical characteristics at baseline of CKD patients (n = 20)

Variable	Values[a]
Age	55.10 ± 2.65
Males (%)	55%
Systolic BP (mmHg)	128.6 ± 3.68
Diastolic BP (mmHg)	83.85 ± 3.60
MAP (mmHg)	84.35 ± 4.21
Scr (mg/dL)	1.30 ± 0.09
eGFR (mL/min per 1.73 m^2)	79.25 ± 3.79
BUN (mol/L)	5.4 ± 0.24

Abbreviations: Systolic BP，Systolic blood pressure；Diastolic BP ，Diastolic blood pressure ；MAP ，mean aterialpressure ；eGFR， estimated glomerular filtration； Scr， serum creatinine；BUN ，Blood urea nitrogen.

[a] data are presented as mean ± SEM.

Table S7. Body weight of mice in each group after 14 days of modeling

Group	Body weight (g)	P value
Sham	23.27 ±0.4346	0.6005
UUO + Saline	22.25 ±0.6785	
UUO + Har 10 mg/kg	21.21 ±0.5466	0.2535
UUO + Har 20 mg/kg	20.85 ±0.5004	0.1190

*P-value < 0.05 compared with UUO + Saline determined by two-tailed t test.

Review Files

Figure 2H

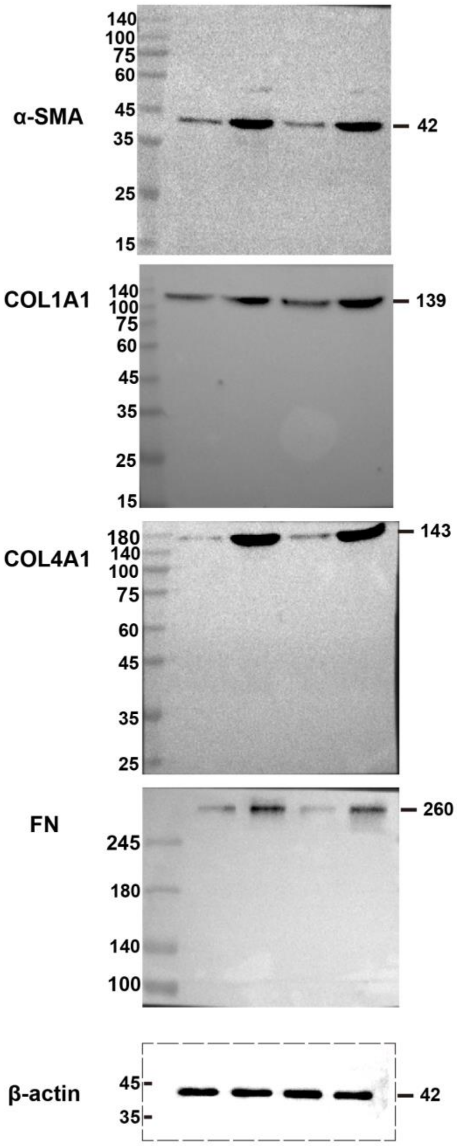

Figure 4B

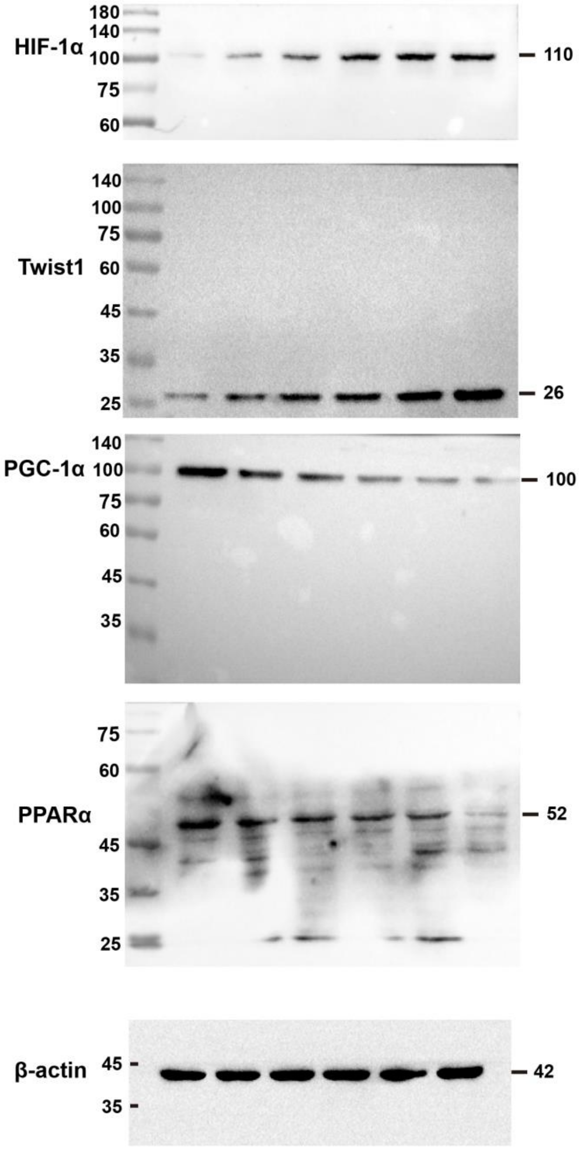

Figure 5A

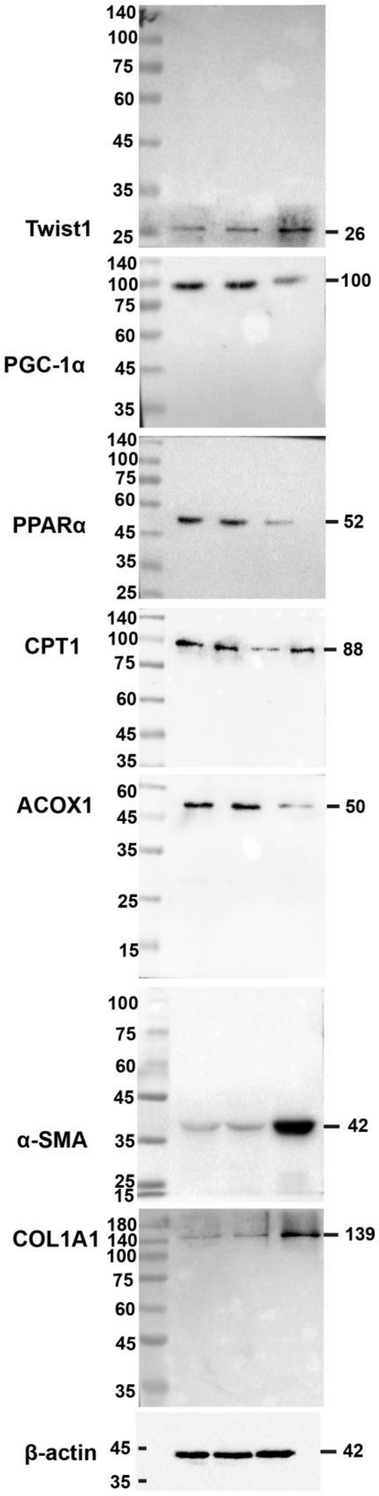

Figure 5B

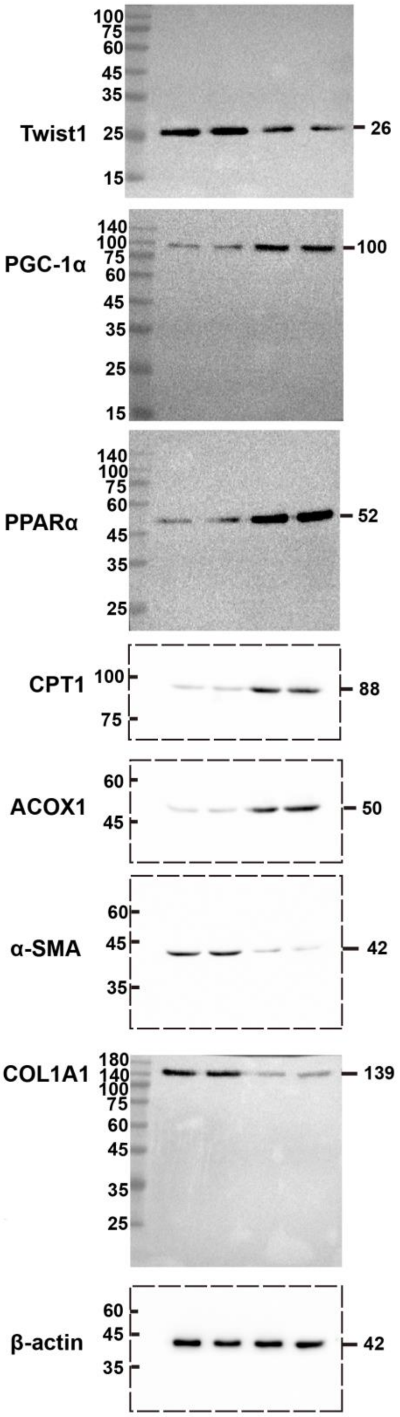

Figure 5G

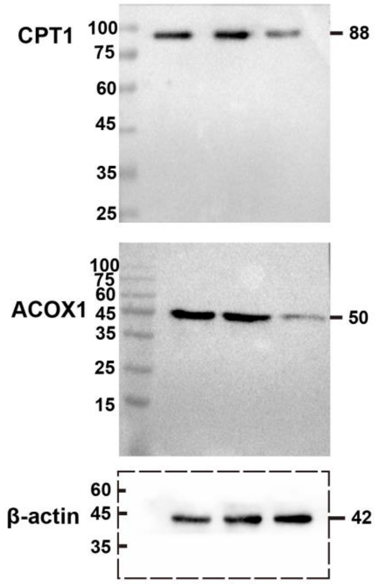

Figure 5i

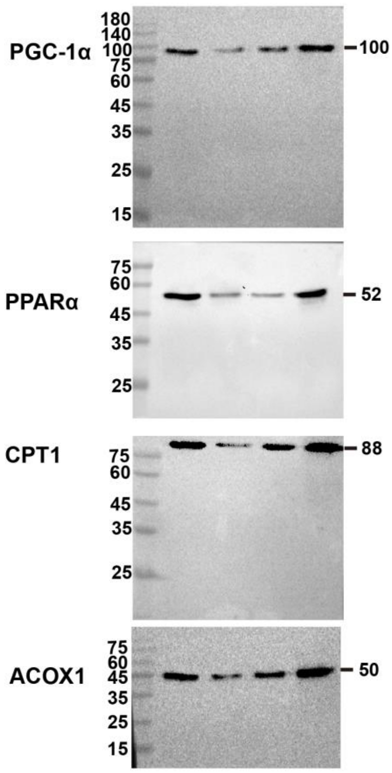

Figure 5I

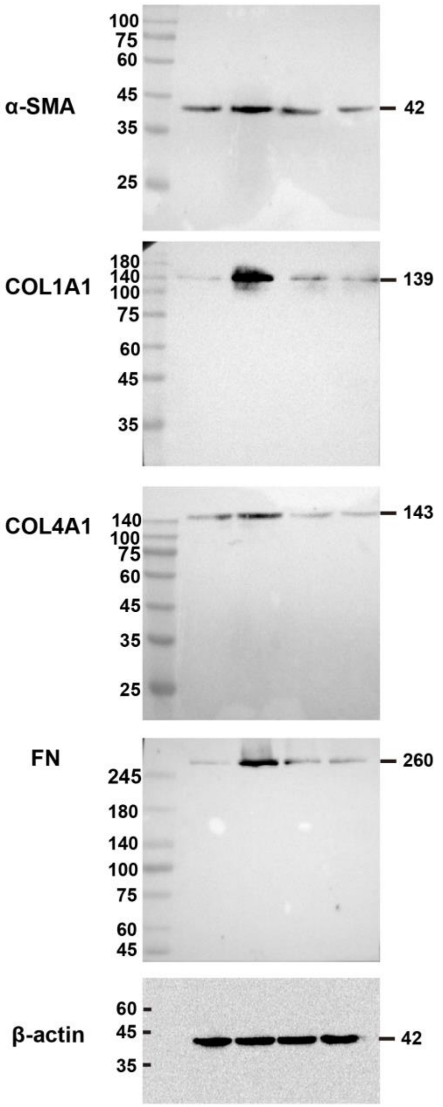

Figure 6A

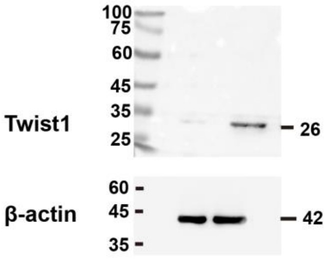

Figure 6H

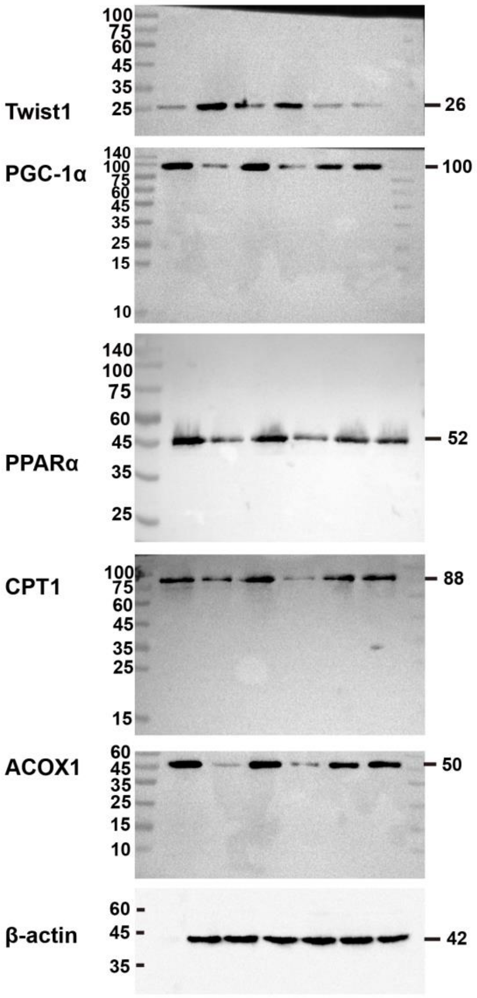

Figure 6I

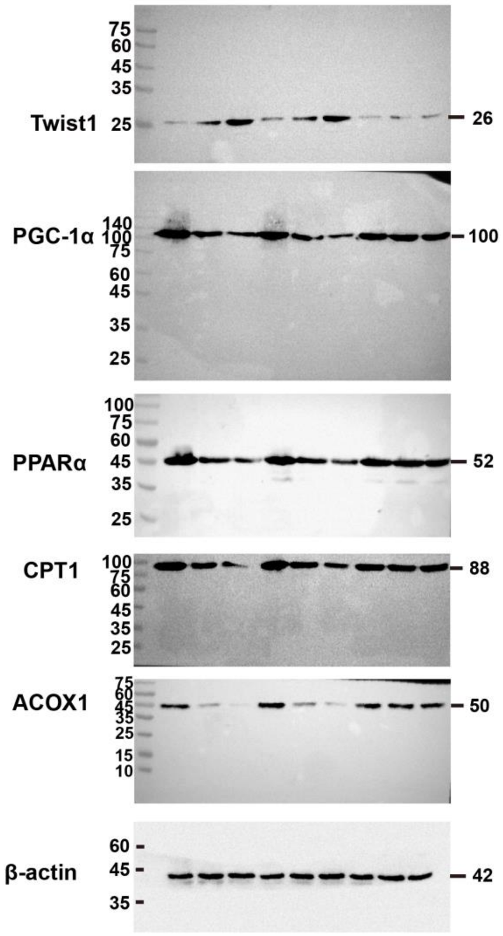

Figure 7G

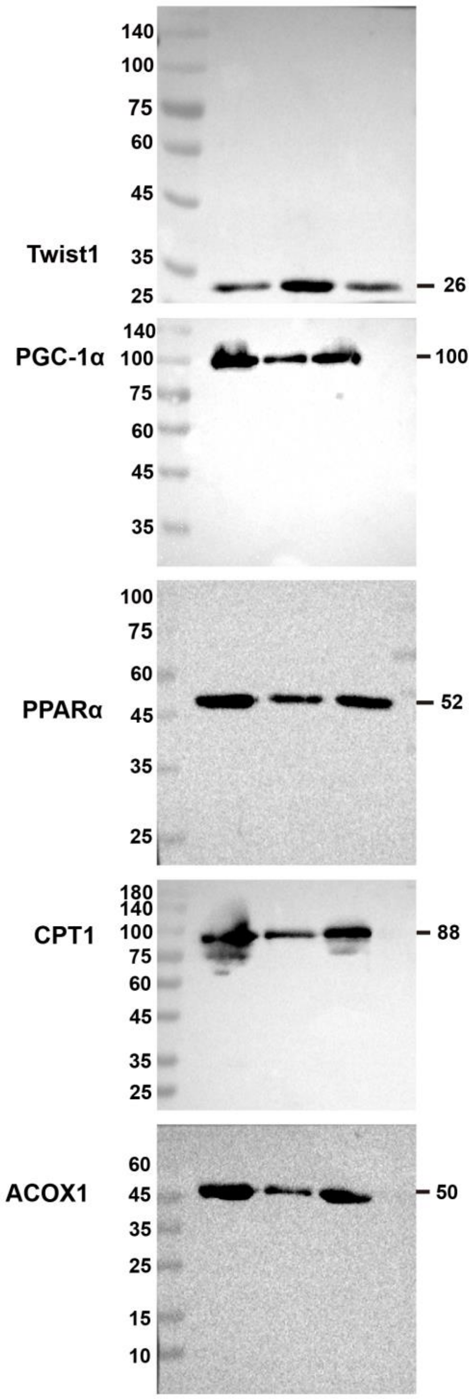

Figure 7G

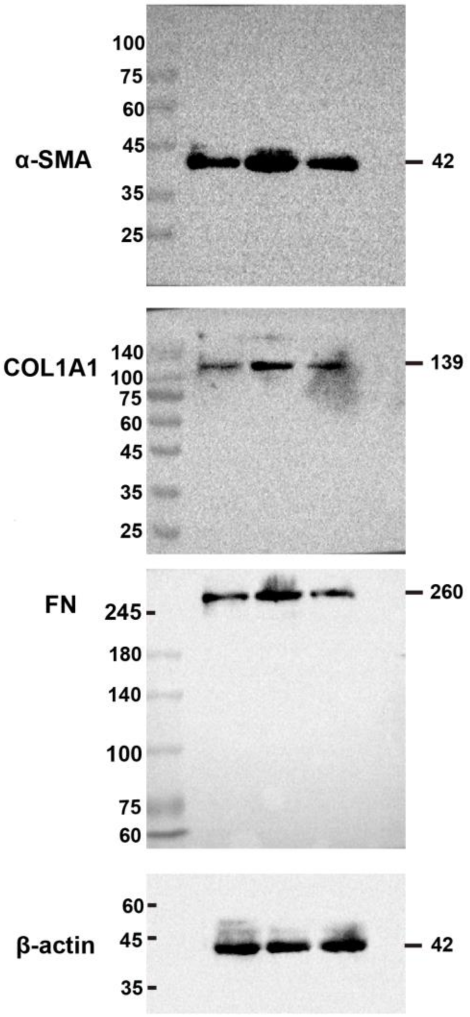

Supplementary Figure 3

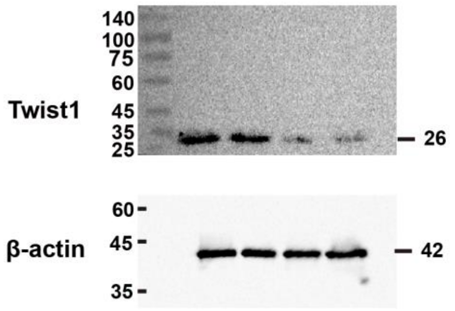

Supplement: Supplementary file 1 — Supplementary figures and tables. [file thnov12p3758s1.pdf]
